# Androgen receptor imprints satellite cell stemness and preserves their reservoir for lifelong regeneration and optimal repair

**DOI:** 10.1126/sciadv.aec8073

**Published:** 2026-09-09

**Authors:** Joe G. Rizk, Kamar C. Ghaibour, Sirine Souali-Crespo, Emilia Calvano, Aurore Bilger, Hugues Jacobs, Nadia Messaddeq, Qingshuang Cai, Erwan Grangirard, Rajesh Sahu, Arnaud Ferry, Valentin Fourcade, Joffrey Zoll, Gianni Zanardelli, Nacho Molina, Coralie Fontaine, Jean-François Arnal, Daniel Metzger, Delphine Duteil

**Affiliations:** ^1^ IGBMC UMR 7104- UMR-S 1258, Université de Strasbourg, CNRS, Inserm, F-67400 Illkirch, France.; ^2^ Centre de Recherche en Myologie, UMRS974, Sorbonne Université, INSERM U974, Association Institut de Myologie, F-75013 Paris, France.; ^3^ Biomedicine Research Center of Strasbourg (CRBS), UR 3072, University of Strasbourg, University Hospital of Strasbourg, F-67000 Strasbourg, France.; ^4^ Institut National de la Santé et de la Recherche Médicale (INSERM), U1297, Institut des Maladies Métaboliques et Cardiovasculaires (I2MC), Université Toulouse III Paul Sabatier, CHU Rangueil (University Hospital) de Toulouse, F-31432 Toulouse, France.

## Abstract

Skeletal muscle stem cells (MuSC) are the guardians of muscle regeneration, sustaining tissue integrity through a delicate balance of quiescence, activation, and lineage commitment. While numerous molecular cues have been implicated in regulating these processes, the influence of androgen receptor (AR) signaling, an essential hormonal pathway for male muscle physiology, has remained largely unexplored. Here, we show that AR expression defines quiescent MuSC and acts as a safeguard of their dormancy. Integrated multiomic analyses reveal a redistribution of AR binding from quiescence-maintenance loci to regulatory elements driving activation and metabolic reprogramming during repair. Loss of AR in young adult mice disrupts this balance, precipitating premature cell-cycle entry, skewed division modalities, depletion of the stem cell reservoir, and destabilization of the niche. These defects converge with hallmarks of aging-associated androgen decline, while androgen supplementation restores regenerative competence. Together, our findings establish AR signaling as a pivotal determinant of MuSC fate and a cornerstone of skeletal muscle homeostasis.

## INTRODUCTION

Skeletal muscles exhibit a regeneration capacity upon injury, resulting from the fusion of newly formed myocytes to generate myofibers that replace the damaged ones. This process is initiated by myofiber degeneration, causing inflammatory cell infiltration followed by the activation of satellite cells, which are the muscle stem cells (MuSC) (*1*). In the intact muscle, MuSC reside in a quiescent state within a specialized niche between the myofiber sarcolemma and the basal lamina (*2*, *3*). They are defined by the expression of the myogenic transcription factor paired box 7 (*Pax7*), a key marker of their identity and function (*4*).

Upon muscle injury, MuSC exhibit a dual capacity. They can either self-renew to replenish the stem cell pool and preserve long-term regenerative competence or enter a proliferative phase followed by activation of the myogenic differentiation program, culminating in fusion and formation of new myofibers (*5*, *6*). These key myogenic features are intrinsically dependent on MuSC microenvironment, comprising immune and stromal cells. This microenvironment is tightly regulated by interactions with extracellular matrix (ECM) components, intercell signaling, and paracrine or endocrine stimuli (*7*–*9*). With age, skeletal muscle regenerative capacities progressively decline, which has been recently attributed to a reduction in MuSC number and function (*10*–*12*). These processes are thought to be exacerbated by hormonal changes, including decrease in circulating androgens (*13*–*15*).

Androgens are steroid hormones mainly produced by male gonads. They play a crucial role in modulating muscle homeostasis and MuSC fate, both during postnatal development and at adult stages (*16*, *17*). In young adult mice, androgens promote MuSC quiescence through a Notch-dependent pathway (*18*) and influence their activation, proliferation, and differentiation (*18*–*20*). In addition, androgen supplementation increases MuSC numbers in both animal models and humans (*21*). Oppositely, castration-induced androgen deprivation in rodents leads to perturbed MuSC homeostasis (*22*). Androgens exert their effects through the androgen receptor (AR), a ligand-activated transcription factor and member of the nuclear receptor superfamily, which upon ligand binding translocates to the nucleus and binds specific DNA sequences, known as androgen response elements (AREs), to regulate target genes expression (*23*–*25*). We previously showed that AR in myofibers is instrumental for the musculoskeletal system by controlling gene networks required for structural and metabolic demands (*25*, *26*). Nevertheless, the role of androgens via AR in MuSC during muscle regeneration, the broader consequences of androgen-dependent decline on the regenerative machinery, the underlying molecular mechanisms, and its potential causal link to age-associated muscle degeneration remain to be fully elucidated.

By integrating genome-wide profiling and histological analysis, with the study of an inducible MuSC-specific AR knockout mouse model and stemlike myogenic cell lines, we delineate the molecular mechanisms through which androgens via their receptor regulate MuSC regenerative functions. Our findings highlight the pivotal role of MuSC-AR in muscle repair, identifying AR-dependent stem cell profiles and supportive MuSC subtypes involved in postnatal myogenesis. We further reveal that AR orchestrates stemness transitions by sequential chromatin repositioning following injury, thereby controlling gene networks governing MuSC self-renewal, proliferation, and differentiation. In addition, we demonstrate that androgen signaling contributes to the age-related decline in muscle healing capacities, and validate the translational relevance of murine findings in human myoblasts.

## RESULTS

### AR deficiency in MuSC in young individuals compromises regenerative capacity and alters metabolic and structural transcriptional programs in skeletal muscle

To dissect the age-dependent role of AR signaling within MuSC, we generated mice with a conditional AR deletion in this cell lineage (hereafter AR^(i)sat-/Y^ mice). Nine-week-old mice harboring floxed *Ar* alleles and expressing the *Pax7*-CreER^T2^ transgene were treated with tamoxifen (TAM) for 5 days, enabling selective deletion of *Ar* in MuSC (fig. S1, A to D). TAM-treated littermates lacking the *Pax7*-CreER^T2^ transgene served as controls. To evaluate the functional consequences of AR ablation on skeletal muscle regenerative capacities, we subjected young (3-month-old) control and AR^(i)sat-/Y^ mice to acute tibialis anterior (TA) muscle injury via cardiotoxin (CTX) injection 1 month after gene ablation, and assessed histological features of repair at 5, 7, 14, 28, and 42 days postinjury (dpi).

**Fig. 1.**
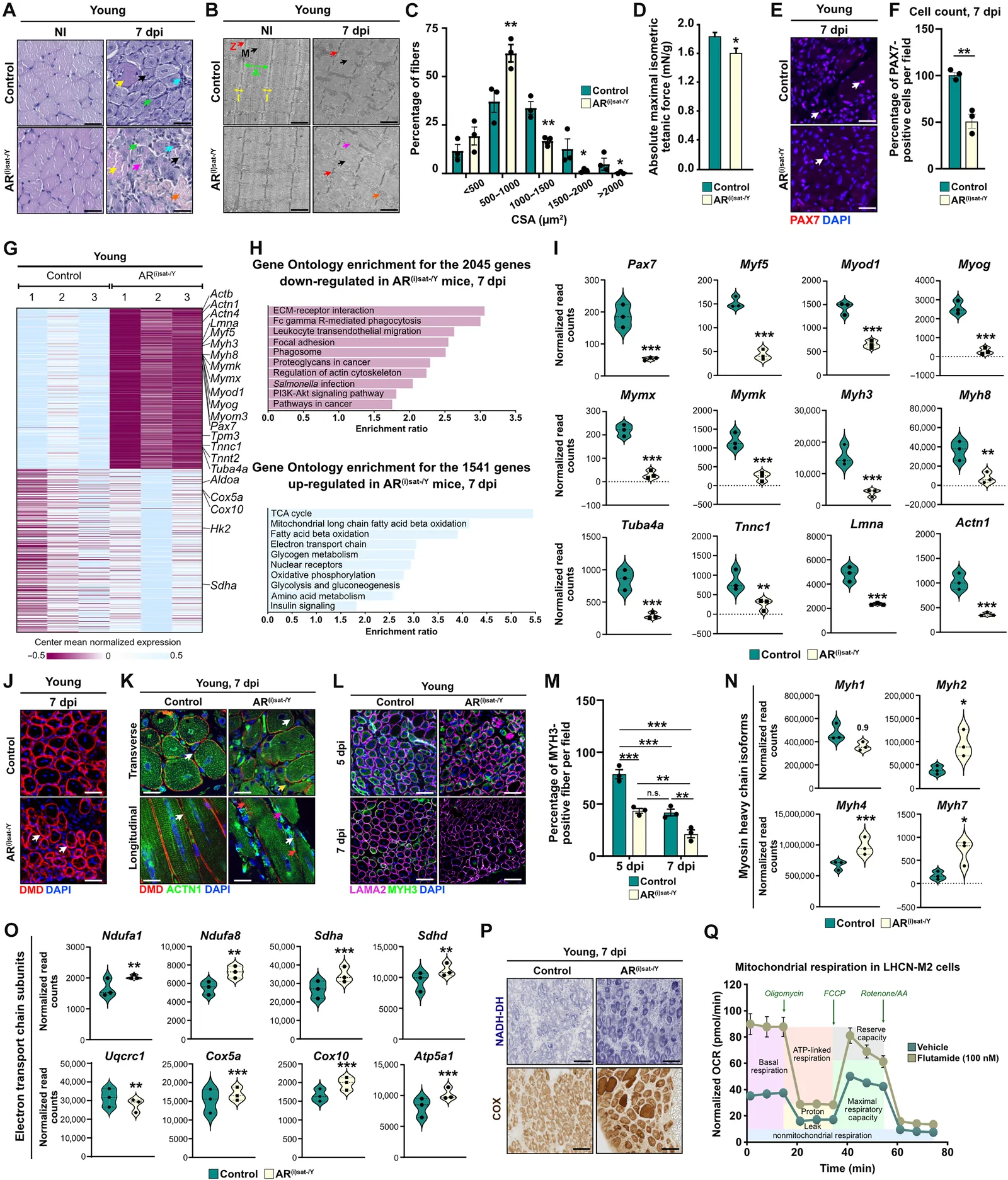
Absence of MuSC-AR impairs muscle regenerative capacities at young age. (**A**) Representative hematoxylin and eosin (H&E) staining of tibialis anterior (TA) muscles of noninjured (NI) control and AR^(i)sat-/Y^ mice and at 7 dpi. Arrows: black, centronucleated fibers; cyan, immune infiltration; yellow, necrotic fibers; green, interstitial space; pink, misshaped regenerating myofibers; orange, fibrosis. (**B**) Ultrastructure analysis of TA of control and AR^(i)sat-/Y^ mice. Z-, M-, I-, and A-bands are pointed. Arrows: black, M-line disturbances; red, Z-line disruption; pink, T-tubules misalignments; orange, fibrosis. (**C**) Myofiber CSA of control and AR^(i)sat-/Y^ mouse TA 7 dpi. (**D**) In situ single-fiber isometric force of 28-day–injured control and AR^(i)sat-/Y^ mouse TA. (**E** and **F**) Immunofluorescence of PAX7 (red) (E), and corresponding quantification (F) in control and AR^(i)sat-/Y^ mouse TA 7 dpi. White arrows: PAX7-positive cells. 4′,6-diamidino-2-phenylindole (DAPI): nuclei. (**G** to **I**) Heatmap of mean centered normalized expression of indicated genes in TA of control and AR^(i)sat-/Y^ mice 7 dpi (G), corresponding pathway analysis (H), and violin plots (I). TCA, tricarboxylic acid. (**J** and **K**) DMD (red) and ACTN1 (green) immunofluorescence in control and AR^(i)sat-/Y^ mouse TA 7 dpi. DAPI: nuclei. (J) White arrows: fibers within other fibers. (K) Arrows: white, nuclei; yellow, sarcolemma discontinuation; red, ACTN1 aggregates; pink, stair shape. (**L** and **M**) MYH3 (green) and LAMA2 (magenta) immunofluorescence (L), and corresponding quantification of MYH3-positive cells (M) in 5- and 7-day–injured TA of control and AR^(i)sat-/Y^ mice. DAPI: nuclei. (**N** and **O**) Normalized read counts of the indicated genes in control and AR^(i)sat-/Y^ mouse TA 7 dpi. (**P**) Histochemistry of nicotinamide adenine dinucleotide (NADH)–dehydrogenase (DH) and cytochrome c oxidase (COX) activities in control and AR^(i)sat-/Y^ mouse TA 7 dpi. (**Q**) Mitochondrial respiration of vehicle and 100 nM flutamide-treated LHCN-M2 cells, assessed by Seahorse Mito Stress Test. AA, antimycin A. Scale bar, 75 μm [(A) and (L)], 1 μm (B), 50 μm [(E) and (K)], 100 μm (J), and 200 μm (P). Data are mean ± SEM. Statistical tests: (C) two-way analysis of variance (ANOVA) with Šidák’s correction, [(D), (F), and (Q)] two-tailed Mann-Whitney, (M) two-way ANOVA with Tukey’s correction, and [(I), (N), and (O)] Wald test. n.s., not significant; *P < 0.05; **P < 0.01; ***P < 0.001.

While no gross phenotypical abnormalities were noticed in the absence of injury (fig. S1, E to G), both genotypes showed similar hallmarks of effective regeneration, including centronucleated fibers, necrosis, and immune cell infiltration at 5 dpi (fig. S1H), a time point at which MuSC activation and early differentiation culminate (*27*). However, at 7 dpi, AR^(i)sat-/Y^ mice exhibited regenerative defects, including persistent inflammation, and aberrant fiber morphology that persisted until 42 dpi and were not due to the CreER^T2^ knock-in (Fig. 1A and fig. S1H). Ultrastructural analyses performed at 7 dpi, a time point at which mononuclear cells fuse to form myosyncytia and primary regenerative outcomes are captured (*28*), revealed that AR^(i)sat-/Y^ muscles displayed disorganized sarcomeres, with misaligned triads characterized by dilated T-tubules, disrupted Z-lines, and poorly defined or absent M-lines (Fig. 1B). These structural muscle defects correlated with a significant reduction in myofiber cross-sectional area (CSA; Fig. 1C), which translated into a significant reduction in contractile force at 28 dpi (Fig. 1D). Muscle dysfunction was accompanied by a marked reduction in the MuSC pool at 7 dpi (Fig. 1, E and F), which persisted at 28 dpi (fig. S1, I and J), showing that loss of AR in MuSC severely impairs regenerative capacity in young male mice.

To elucidate AR transcriptional landscape, we conducted bulk RNA sequencing (RNA-seq) on TA muscles harvested at 7 dpi from young AR^(i)sat-/Y^ and control mice. Our analysis revealed a differential expression of 3586 genes, comprising 2045 down-regulated and 1541 up-regulated genes in AR-deficient mice (Fig. 1G and fig. S1K). Pathway analyses of the down-regulated genes highlighted a strong association with biological processes involved in actin cytoskeleton organization, focal adhesion, and developmental signaling cascades such as the phosphatidylinositol 3-kinase (PI3K)–Akt pathway (Fig. 1H). Notably, the expression of key myogenic regulators, including *Pax7*, *Myf5*, *Myod1*, *Myog*, and fusion-mediating factors such as *Myomixer* (*Mymx*), and *Myomaker* (*Mymk*), was markedly reduced in AR^(i)sat-/Y^ muscles (Fig. 1I), in agreement with the decreased MuSC number. Similarly, transcripts encoding contractile cytoskeletal components such as actin, actinin, troponin and tropomyosin isoforms (e.g., *Actb*, *Actn1*, *Actn4*, *Tnnc1*, *Tnnt2*, and *Tpm3*), and embryonic myosin heavy chain isoforms (*Myh3*, embryonic and *Myh8*, perinatal), as well as noncontractile components, including laminins (*Lmna*), *Myomesin 3* (*Myom3*), and *Dystrophin* (*Dmd*), were substantially diminished (Fig. 1, G and I), providing a molecular rationale for the morphological abnormalities observed at this time point. In agreement, immunofluorescence analysis of DMD at 7 dpi revealed profound structural abnormalities in AR^(i)sat-/Y^ mice compared to control littermates (Fig. 1J). Moreover, costaining for sarcomeric alpha actinin (ACTN1) and DMD on transverse sections showed disrupted sarcomere alignment and uneven ACTN1 distribution in mutant fibers, while longitudinal sections confirmed major striation defaults (Fig. 1K, pink arrows). Notably, whereas healing control fibers had centrally positioned nuclei, AR^(i)sat-/Y^ fibers showed disorganized central nuclear localization (Fig. 1K, white arrows), reflecting a disrupted cytoskeletal organization. Consistent with our transcriptomic data, immunostaining for embryonic myosin heavy chain (MYH3) at 5 and 7 dpi showed a ∼50% reduction in the number of MYH3-positive fibers in AR^(i)sat-/Y^ mice compared to controls (Fig. 1, L and M), signifying delayed or incomplete myogenic progression. To further investigate this impairment, we examined the expression profile of myosin heavy chain isoforms (Fig. 1N). In contrast to the down-regulation of *Myh3* and *Myh8*, transcripts of *Myh2*, *Myh4*, and *Myh7* (encoding MyHC IIa, IIb, and I, respectively) were up-regulated (Fig. 1N), reflecting a shift toward increased expression of adult myosin heavy chain isoforms at the expense of fetal ones, although *Myh1* (MyHC IIx) did not reach statistical significance (Fig. 1N). Transcriptomic analysis of the 1541 genes up-regulated in 7 dpi–injured AR^(i)sat-/Y^ TA muscles revealed a predominant enrichment in metabolic pathways, particularly those governing glycolysis, oxidative phosphorylation, mitochondrial function, the metabolism of glycogen, fatty acids, and amino acids (Fig. 1H). Notably, expression of several key regulators of cellular metabolism, including aconitase 2 (*Aco2*), citrate synthase (*Cs*), and multiple isoforms of isocitrate dehydrogenase (e.g., *Idh2* and *Idh3a*), was markedly elevated in AR^(i)sat-/Y^ muscles. This was paralleled by increased expression of genes and components of the oxidative phosphorylation machinery, including most of the members of mitochondrial complex I (e.g., *Ndufa1*–*13*), II (e.g., *Sdha*-*d*), III (e.g., *Uqcrc1*–*2*), IV (e.g., *Cox5a* and *Cox10a*), and V (e.g., *Atp5a1* and *Atp5b*) (Fig. 1O). Notably, the expression of these genes was not affected in the absence of injury (fig. S1, L and M).

To investigate whether the absence of MuSC-AR during regeneration promotes a metabolic shift toward a more oxidative muscle fiber phenotype, we stained nicotinamide adenine dinucleotide (NADH) dehydrogenase activity (NADH-DH) 7 dpi. In regenerating TA muscles from control mice, we observed lightly stained fibers, indicative of glycolytic metabolism. In contrast, AR^(i)sat-/Y^ muscles displayed a higher presence of darkly stained fibers (Fig. 1P), reflecting increased NADH-DH activity. These findings were corroborated by cytochrome c oxidase (COX) activity staining, indicative of a glycolytic-to-oxidative shift in fiber-type composition (Fig. 1P). These findings prompted us to investigate whether such metabolic alterations are intrinsically rooted in MuSC. Seahorse analysis performed on LHCN-M2 human myoblasts, treated with 100 nM of the AR antagonist flutamide, unveiled an increased oxidative phosphorylation activity when compared to vehicle-treated cells (Fig. 1Q and fig. S1N), indicating that the metabolic abnormalities observed in regenerating myofibers may originate from intrinsic defects in MuSC. In parallel, basal ECAR was measured for glycolysis analyses (fig. S1, O and P). Together, our data show that AR loss in young individuals leads to MuSC depletion and important structural defects, associated with a fiber-type switch toward oxidative metabolism.

### Androgen signaling in MuSC coordinates gene programs essential for homeostasis and regeneration

**Fig. 2.**
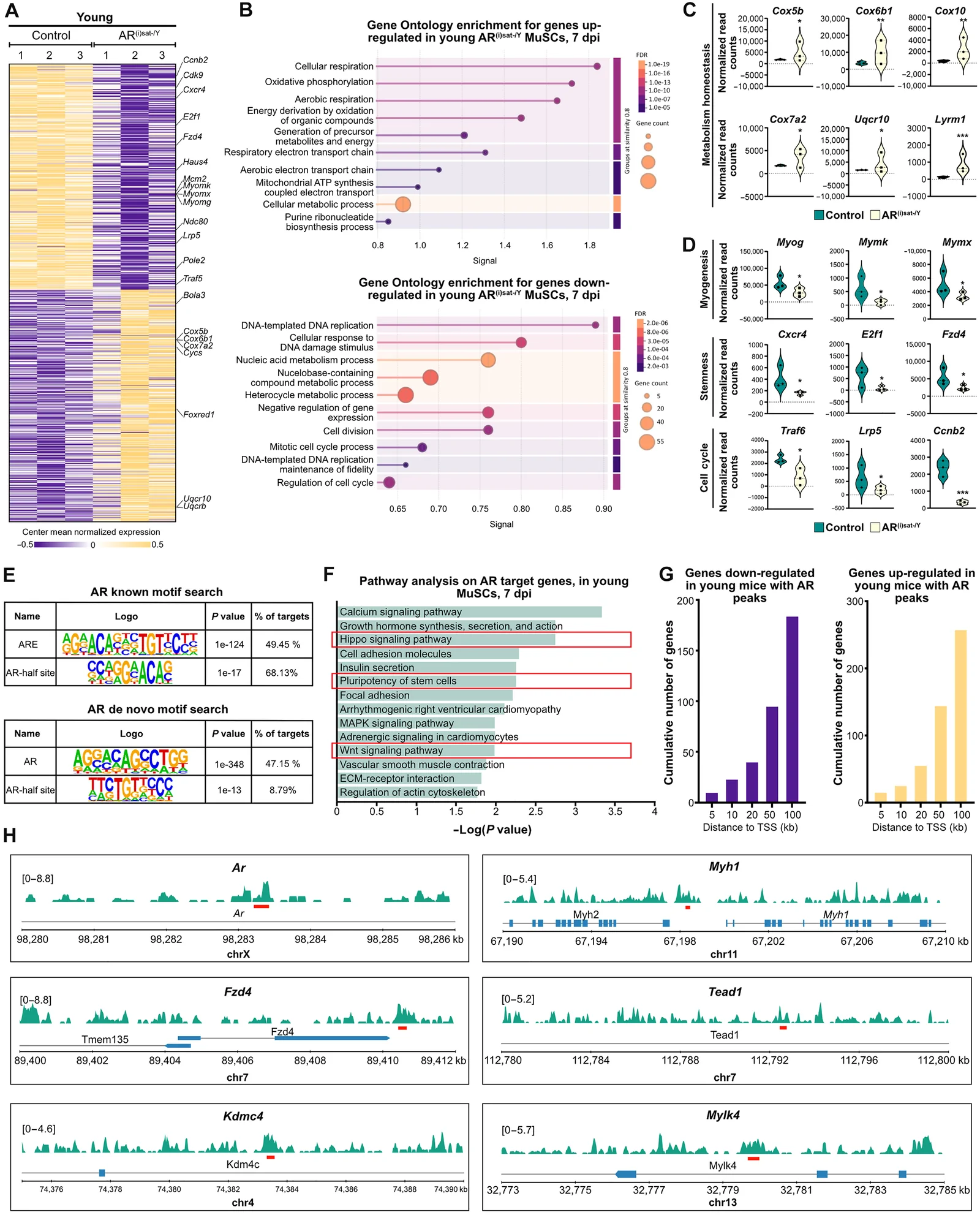
Characterization of AR cistrome and transcriptome in MuSC at young age. (**A**) Heatmap depicting the mean centered normalized expression of indicated genes selected from bulk RNA-seq analysis performed in 7-day–injured TA of young control and AR^(i)sat-/Y^ male mice. (**B**) Pathway analysis of down- and up-regulated genes in 7-day–injured TA of young control and AR^(i)sat-/Y^ male mice. (**C** and **D**) Violin plots representing the normalized read counts of the indicated genes, selected from bulk RNA-seq analysis performed in 7-day–injured TA of young control and AR^(i)sat-/Y^ male mice. Statistical test used is Wald test. *P < 0.05; **P < 0.01; ***P < 0.001. (**E**) HOMER known and de novo motif analyses of AR-bound DNA sequences from 7-day–injured TA of young male mice. (**F**) Pathway analysis performed on AR target genes identified by CUT&RUN in MuSC of 7-day–injured TA of young male mice. Pathways of interest are squared in red. MAPK, mitogen-activated protein kinase. (**G**) Cumulative number of down-regulated genes and up-regulated genes in MuSC of 7-day–injured TA of young male mice, with an AR peak located at the indicated distances from their transcription start site (TSS). (**H**) AR localization at the *Ar*, *Fzd4*, *Kdmc4*, *Myh1*, *Tead1*, and *Mylk4* loci, with binding sites shown in red.

To elucidate the cell-specific AR–dependent molecular mechanisms governing MuSC homeostasis during skeletal muscle regeneration, we performed a SMART-seq on fluorescence-activated cell sorting (FACS)–isolated MuSC from 7 dpi–injured TA of young (15-week-old) control and AR^(i)sat-/Y^ male mice. Transcriptomic analysis revealed differential expression of 821 genes in AR-deficient MuSC, including 327 down- and 494 up-regulated genes (Fig. 2A and fig. S2A). Consistent with the Seahorse assay findings and the histological evidence of a metabolic shift toward oxidative metabolism at 7 dpi, analysis of the up-regulated genes highlighted a strong enrichment of pathways related to metabolic processes, particularly cellular respiration and oxidative phosphorylation (Fig. 2B). In particular, the expression of *Cox5b* and *Cox6b1*, in addition to numerous genes implicated in mitochondrial homeostasis, was significantly elevated in AR^(i)sat-/Y^ MuSC (Fig. 2C). Conversely, pathway analysis of the down-regulated gene set revealed a significant association with cell cycle regulation (e.g., DNA replication and cell division) (Fig. 2, B and D).

To identify the direct transcriptional targets of AR, we isolated MuSC from TA muscles of control mice by FACS at 7 dpi and performed CUT&RUN assays using AR-specific antibodies, alongside profiling of demethylated histone H3 (H3K4me2), a well-established marker of active promoters and enhancers. Bioinformatic analysis identified 7346 AR-binding peaks, predominantly located within intronic and intergenic regions encompassing the canonical AREs (Fig. 2E and fig. S2B), as well as 39,900 peaks associated with the H3K4me2 mark, evenly distributed between promoter, intron, and intergenic regions (fig. S2C). Integrative analysis of the two datasets revealed that AR is recruited to 3104 transcribed genes (fig. S2D). Gene Ontology and pathway enrichment analyses of AR-bound genes uncovered a strong association with key developmental and regenerative pathways, including the HIPPO (e.g., *Tead1*, *Wwc1*, and *Fat4*) and WNT signaling cascades (e.g., *Wnt5a* and *Fzd4*), as well as signaling modules related to tissue remodeling and regeneration, such as cell adhesion, focal adhesion, ECM-receptor interactions, and actin cytoskeleton regulation (Fig. 2F). Notably, AR occupancy was also observed at genes essential for stem cell homeostasis (e.g., *Foxo1* and *Bcl2*), muscle repair (e.g., *Mstn*, *Cxcl2*, and *Cdh15*), and sarcomere assembly or function (e.g., *Myh1*, *Mylk4*, and *Myom2*). A transcription start site (TSS) gene-centric analysis demonstrated that AR binds more than the two-thirds of both down- and up-regulated genes in AR^(i)sat-/Y^ mice within a 100-kb window (Fig. 2G), showing that AR predominantly regulates gene expression through distal elements. Representative AR binding loci within key genes involved in the aforementioned pathways, including *Ar* itself, *Fzd4*, *Kdmc4*, *Myh1*, *Tead1*, and *Mylk4*, are illustrated in Fig. 2H. Gene Ontology analysis revealed that genes down-regulated in association with AR peaks at 7 dpi were predominantly involved in cellular structural and organelle-related processes (fig. S2E), whereas up-regulated genes were enriched in metabolic pathways (fig. S2F), thereby providing a molecular basis for the impaired phenotype observed at the histological level. Together, these findings support a model in which AR orchestrates regenerative transcriptional programs in MuSC via enhancer-driven mechanisms.

### Single-cell RNA-seq reveals early molecular and cellular alterations in the AR-deficient MuSC microenvironment

**Fig. 3.**
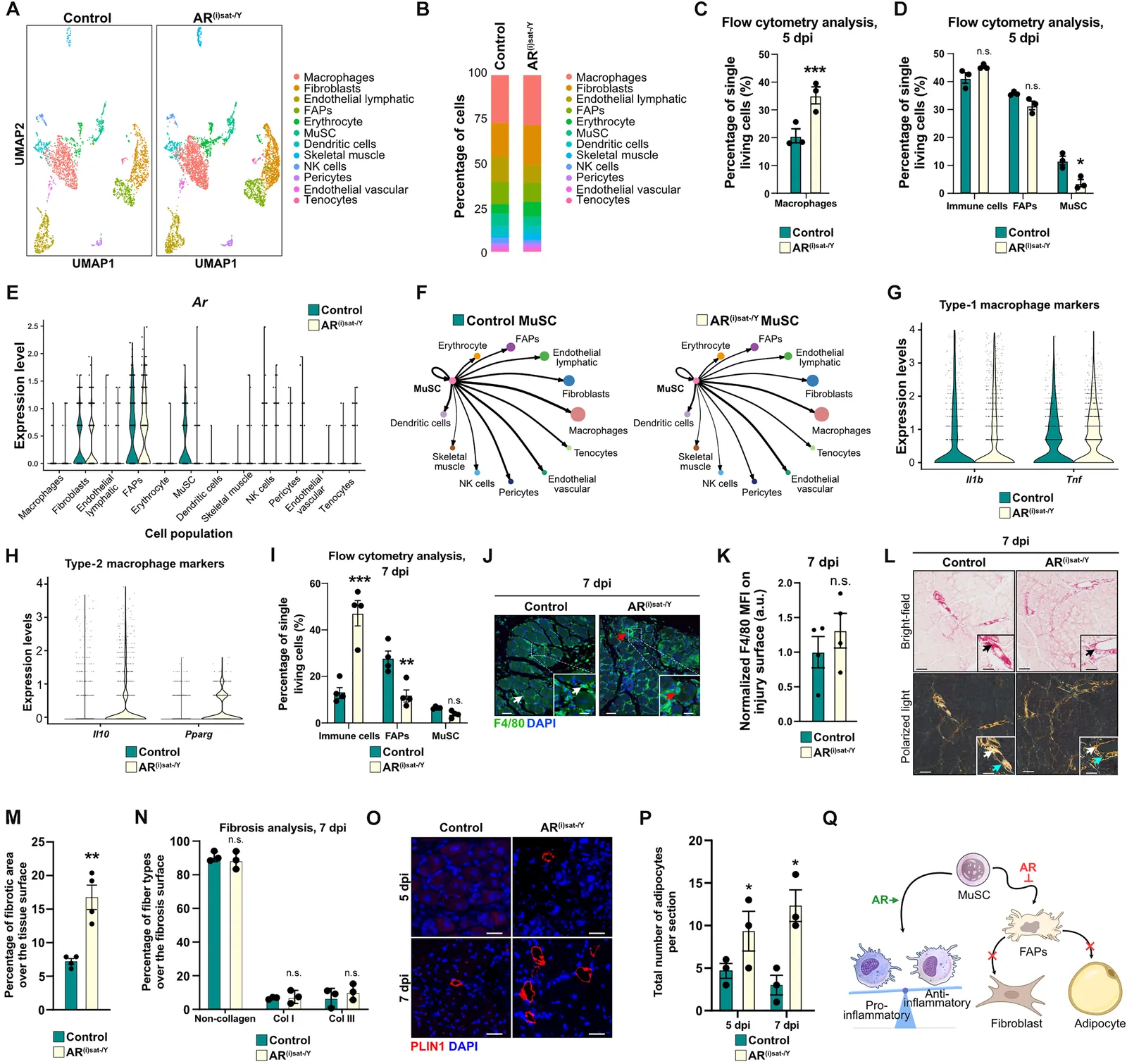
MuSC microenvironment is altered upon AR loss. (**A** and **B**) Single-cell uniform manifold approximation and projection (UMAP) (A), and respective cell-type percentage (B) from 5-day–injured control and AR^(i)sat-/Y^ mouse TA. (**C** and **D**) Percentage of macrophages (C), immune cells, and FAPs and MuSC (D) in 5-day–injured control and AR^(i)sat-/Y^ mouse TA from flow cytometry analysis. (**E**) *Ar* transcript levels in indicated cell populations from 5-day–injured control and AR^(i)sat-/Y^ mouse TA. (**F**) Scheme of cell-cell communication between MuSC and other TA constitutive cells types in 5-day–injured control and AR^(i)sat-/Y^ mouse TA. Arrow thickness: relative interaction strength between populations. (**G** and **H**) Transcript levels of pro- (G) and anti-inflammatory macrophage markers (H) in 5-day–injured control and AR^(i)sat-/Y^ mouse TA. (**I**) Percentage of immune cells, FAPs, and MuSC, in 7-day–injured control and AR^(i)sat-/Y^ mouse TA from flow cytometry analysis. (**J** and **K**) F4/80 immunofluorescence (green) (J) and corresponding quantification of mean fluorescence intensity (MFI) (K) in 7-day–injured control and AR^(i)sat-/Y^ mouse TA. White arrows: interfiber macrophages. Red arrows: intrafiber macrophages. DAPI: nuclei. Scale bars, 100 μm (main) and 30 μm (inset). (**L**) Sirius Red histochemical staining visualized under bright field (top) and polarized light (bottom) in 7-day–injured control and AR^(i)sat-/Y^ mouse TA. Black arrows: collagen fibers. White arrows: type-I collagen; cyan blue arrows: type-III collagen fibers. Scale bars, 250 μm (main) and 90 μm (inset). (**M** and **N**) Quantification of Sirius Red area from bright-field analysis (M), and fibrosis composition (N) of 7-day–injured TA of young control and AR^(i)sat-/Y^ mice. (**O** and **P**) Representative immunofluorescent detection of perilipin-1 (PLIN1; red) (O) and corresponding quantification (P) in 5- and 7-day–injured TA of young control and AR^(i)sat-/Y^ mice. White arrows: adipocytes. DAPI: nuclei. Scale bars, 100 μm. (**Q**) Scheme on AR functions in MuSC within the regenerative niche. Created in BioRender. D.D. (2026); https://BioRender.com/4q7anrd. Data are mean ± SEM. Statistical tests: [(C), (D), (I), (K), (M), and (N)] two-tailed Mann-Whitney and (P) two-way ANOVA with Šidák’s correction. n.s., not significant; *P < 0.05; **P < 0.01; ***P < 0.001.

To decipher the primary events underlying defective MuSC homeostasis caused by AR loss and the consequent impact on their microenvironment during impaired regeneration, we performed single-cell RNA sequencing (scRNA-seq) in 5-dpi–injured TA muscles of young (15-week-old) control and AR^(i)sat-/Y^ mice (fig. S3A). Notably, AR^(i)sat-/Y^ mice expressed the yellow fluorescent protein (YFP) transgene selectively in MuSC to efficiently follow those cells. We identified 12 unsupervised clusters, annotated to the literature (*29*, *30*), based on the expression of specific marker genes (Fig. 3A and fig. S3, A and B), with no evidence of cell population loss or emergence in mutants (Fig. 3, A and B, and tables S1 and S2). However, we observed a slight reduction in the proportion of fibro-adipogenic progenitors (FAPs) and MuSC, as well as an increase in that of macrophages in AR^(i)sat-/Y^ mice compared to controls (Fig. 3B). Flow cytometry analysis of injured TA muscles at 5 dpi furtherly confirmed a ∼30% increase in macrophages number among CD45^+^ immune cells (Fig. 3C), and immunolabeling for F4/80 revealed an upward trend in macrophage abundance (fig. S3, C and D). In parallel, flow cytometry analysis indicated an ∼8% decrease in MuSC, while total immune cells and FAPs were not significantly altered (Fig. 3D). Notably, *Ar* expression was not ubiquitous across all populations; rather, it was predominantly detected in cell types exhibiting stemlike properties or mesenchymal profiles, including FAPs, fibroblasts, and MuSC (Fig. 3E). *Ar* expression was completely abolished in mutant MuSC and not in other cell types (Fig. 3E), while *Yfp* was exclusively expressed in MuSC of AR^(i)sat-/Y^ mice, consistent with the genetic engineering of our model (fig. S3E). Moreover, AR deficiency in MuSC strongly altered the transcriptional profile of their cellular microenvironment (fig. S3F).

To assess intercellular communication within injured muscle tissue at 5 dpi, we used CellChat analysis on scRNA-seq data. In control mice, this analysis revealed a highly interconnected signaling network encompassing all cellular populations, indicative of active and coordinated cross-talk during the regenerative process (fig. S3, G and H). Communication within the MuSC population was diminished in AR^(i)sat-/Y^ mice, as indicated by the decrease in the arrow thickness (Fig. 3F), pointing to a disruption in intrapopulation signaling.

Given the critical interplay between MuSC, macrophages, and FAPs, we next examined the gene networks affected by MuSC-AR loss within these two latter cell populations. Functional enrichment analysis of the 1707 differentially expressed genes (DEGs) upon AR loss in MuSC within macrophages uncovered a transcriptional landscape indicative of macrophage homeostasis (fig. S3I). In particular, transcripts encoding core components of the inflammasome pathway, including caspase 1 (*Casp1*) and its adaptor *Pycard*, were elevated, alongside the chemokines C-C motif chemokine ligand 2 (*Ccl2*) and C-X-C motif chemokine ligand 10 (*Cxcl10*), both of which are known to promote monocyte recruitment. Furthermore, we observed enrichment in metabolic and proteasome pathways intimately linked with immune regulation and tissue remodeling, including oxidative phosphorylation (e.g., *Cox6b1* and *Cox17*), and ubiquitin-proteasome–mediated degradation (e.g., *Uba3*) (fig. S3I). Moreover, macrophage polarization analysis revealed robust expression of canonical proinflammatory markers including *Il1b* and *Tnf* in macrophages from both control and mutant mice at 5 dpi (Fig. 3G). In contrast, macrophages from AR^(i)sat-/Y^ mice expressed anti-inflammatory markers such as interleukin-10 (*Il10*) and peroxisome proliferator–activated receptor gamma (*Pparg*) (Fig. 3H), suggesting a precocious transition toward an anti-inflammatory phenotype in mutant individuals. Further subclustering of the macrophage population revealed that they did not constitute a homogeneous cluster but instead segregated into five transcriptionally distinct subpopulations with specialized functional programs (fig. S3, J to M). Cluster 0 was characterized by high expression of proinflammatory markers such as *Ccr2*, *Il1b*, and *Ccl5*, consistent with recently recruited, monocyte-derived macrophages (fig. S3, J to L). Cluster 1 displayed a strong proinflammatory profile, marked by elevated expression of *Tnf*, *Ccl2*, *Ccl7*, *Cxcl1/2/3*, interferon-stimulated genes (*Ifit1/2/3*), indicative of highly activated macrophages involved in leukocyte recruitment and inflammatory amplification (fig. S3, J to L). Cluster 2 corresponded to phagocytic and lipid-associated macrophages (LAM), expressing canonical LAM markers including *Gpnmb*, *Ctsd*, *Spp1*, *Fabp5*, *Lgals3*, and *Cd36*, suggesting a central role in debris clearance and lipid handling during tissue remodeling (fig. S3, J to L). Cluster 3 was enriched for genes involved in iron metabolism and oxidative stress responses, such as *Slc40a1*, *Hmox1*, *Fth1*, and *Ftl1*, together with *Marco*, *Spic*, and *Arg1*, consistent with specialized macrophages implicated in iron recycling and stress adaptation (fig. S3, J to L). Lastly, cluster 4 exhibited a tissue-resident, reparative phenotype characterized by *Cd163*, *Lyve1*, *Mrc1*, *Retnla*, and *Folr2* expression, alongside ECM and remodeling genes including *Col1a1*/2, *Sparc*, and *Gas6*, indicating anti-inflammatory, proreparative functions (fig. S3, J to L). Differential expression analysis across different populations further revealed cluster-specific functional alterations, with altered antigen-presenting capacity, impaired oxidative metabolism, and shifts toward migratory or scavenger states depending on the subpopulation (fig. S3L). scRNA-seq analysis of macrophage populations revealed an increased representation of an anti-inflammatory macrophage subcluster in AR^(i)sat-/Y^ muscles (fig. S3M). To further examine this observation at the injury site, we performed immunofluorescence labeling for CD163, a marker associated with anti-inflammatory, proreparative macrophages. Quantification revealed an increase in CD163 mean fluorescence intensity (MFI) in AR^(i)sat-/Y^ TA muscles at 5 dpi compared to controls (fig. S3, N and O). These observations are consistent with our scRNA-seq analysis, which identified a macrophage cluster enriched for *Cd163*, *Mrc1*, and *Folr2* expression, indicative of tissue-resident, anti-inflammatory, and remodeling-associated macrophages (fig. S3, J and K).

To determine whether this aberrant immune environment persists over time, we extended our analysis to 7 dpi. Flow cytometry revealed a threefold increase in immune cells in AR^(i)sat-/Y^ TA relative to controls (Fig. 3I), and immunofluorescent labeling of the macrophage-specific marker F4/80 exhibited an increasing trend of the signal [Fig. 3, J (white arrows) and K]. Macrophages were frequently localized inside degenerating muscle fibers of AR^(i)sat-/Y^ mice [Fig. 3, J (red arrows) and K], a phenomenon typically restricted to early regenerative stages, thus indicating delayed resolution of inflammation or prolonged clearance of necrotic tissue. The number of CD163-positive cells was comparable between AR^(i)sat-/Y^ and control muscles, in contrast to the increased signal observed in mutants at 5 dpi (fig. S3, N and O). This temporal pattern may suggest that macrophages in AR^(i)sat-/Y^ muscles transiently acquire reparative features at earlier stages of regeneration. Together, these findings reveal that AR signaling in MuSC plays a pivotal role in modulating the inflammatory response within the injured muscle by affecting the macrophage transcriptomic repertoire.

To unravel the impact of AR loss in MuSC on FAPs’ transcriptional signature, we performed further functional enrichment analysis of their 976 DEGs (fig. S3F). Pathway analysis revealed up-regulation of fibrosis-related transcripts, including *Tgfb*, alongside ECM remodeling enzymes, such as *Adamts* isoforms, *Mmp19*, *Serpine1*, and *Cthrc1*. Moreover, we observed enhanced expression of key adipogenic regulators (*Zeb1*, *Cebpd*, *Socs1*, and *Socs3*). Functional enrichment also highlighted disruptions in metabolic pathways, notably oxidative phosphorylation (*Cox4i1*, *Cox5a*, and *Cox6a1*) and aerobic respiration (*Aco2*, *Hadhb*, *Gls*, and *Lipa*) (fig. S3P), pointing toward a shift in FAPs’ identity, from proregenerative to fibrotic and adipogenic. While FAP numbers were comparable between genotypes at 5 dpi (Fig. 3D), a twofold reduction was observed in AR^(i)sat-/Y^ muscles by 7 dpi (Fig. 3I). We therefore postulated that the decline might be attributed to differentiation of FAPs toward the fibrogenic or adipogenic lineages. To this aim, we performed Sirius Red staining at 7 dpi on injured TA from control and mutant mice. Mutant muscles displayed a marked increase in collagen deposition, with fibrotic area nearly doubled compared to controls (Fig. 3, L and M, black arrows). These findings were corroborated by Masson’s trichrome staining, which revealed a significant enrichment of collagen within the ECM at 7 dpi, with signs of early deposition already evident at 5 dpi in young AR^(i)sat-/Y^ mice (fig. S3Q). Polarized light microscopy revealed that the qualitative composition of the ECM, including collagenous components such as collagens type I [Fig. 3, L (white arrows) and N] and III [Fig. 3, L (cyan blue arrows) and N], and noncollagenous components (e.g., fibronectin, laminins, elastin, tenascin-C, and proteoglycans) remained comparable between genotypes, indicating a quantitative rather than compositional remodeling. We further evaluated adipogenic conversion via perilipin-1 (PLIN1) immunostaining. At 5 dpi, AR^(i)sat-/Y^ muscles already showed early signs of adipocyte emergence, which further increased by 7 dpi, whereas control muscles remained largely devoid of PLIN1-positive cells (Fig. 3, O and P). Thus, our data demonstrate that AR loss in MuSC affects FAP differentiation capacities, promoting fibrosis and adipogenesis. Together, these results indicate that AR signaling in MuSC plays a central role in coordinating the regenerative niche by regulating the balance between pro- and anti-inflammatory immune responses and controlling FAP fate (Fig. 3Q).

### AR deficiency in MuSC at young age perturbs their stemness signatures, disrupts their commitment, and impairs their differentiation potential

**Fig. 4.**
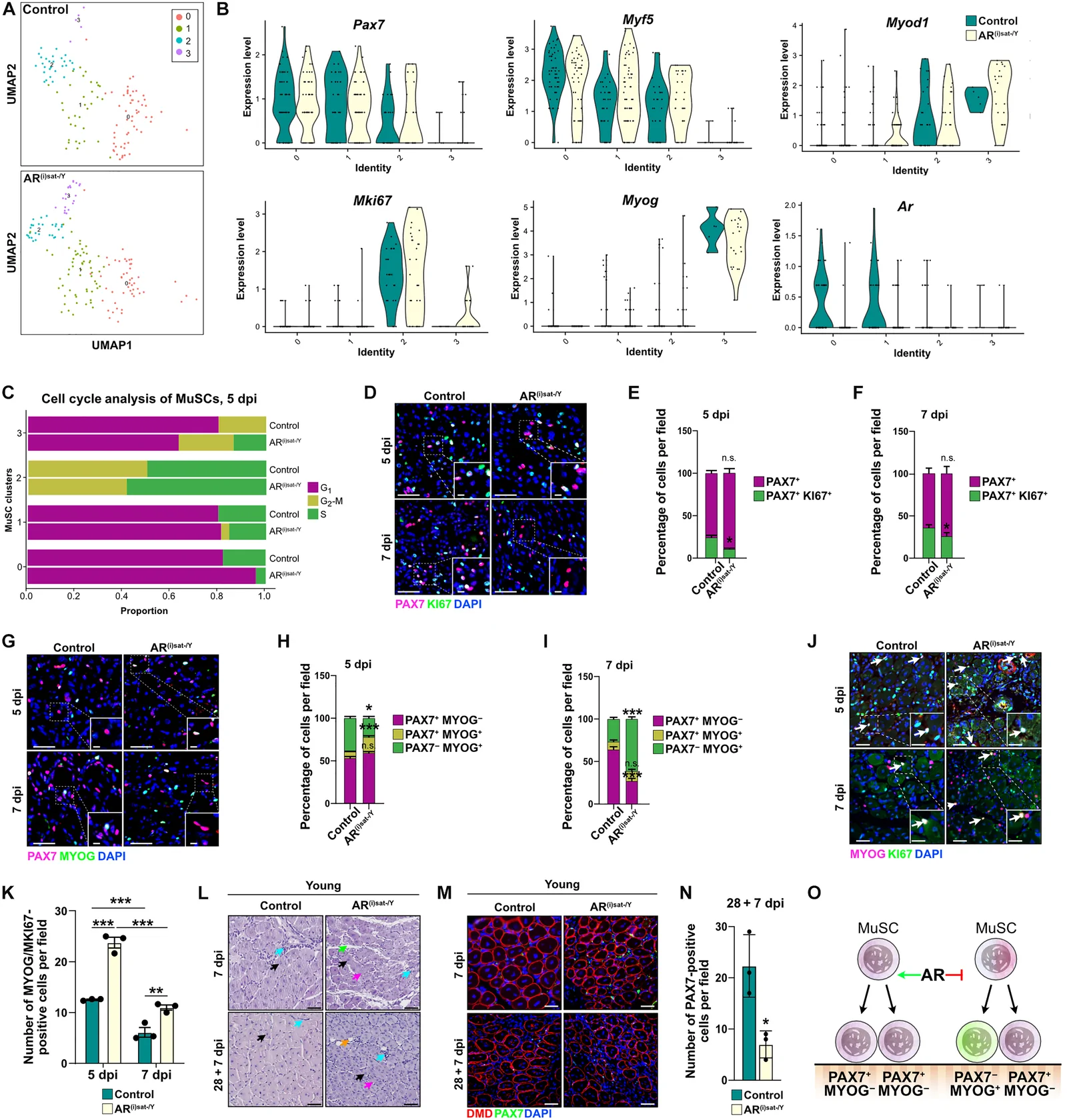
AR in MuSC prevents premature commitment. (**A**) UMAP of MuSC reclusters from 5-day–injured control and AR^(i)sat-/Y^ mouse TA. (**B**) Expression levels of indicated genes in MuSC reclusters, selected from scRNA-seq analysis performed in 5-day–injured control and AR^(i)sat-/Y^ mouse TA. (**C**) Cell cycle distribution across MuSC reclusters in 5-day–injured control and AR^(i)sat-/Y^ mouse TA. (**D** to **F**) Immunofluorescent detection of PAX7 (magenta) and KI67 (green) (D), and corresponding quantifications in 5- (E) and 7-day–injured (F) control and AR^(i)sat-/Y^ mouse TA. DAPI: nuclei. Scale bars, 100 μm (main) and 30 μm (inset). (**G** to **I**) Representative immunofluorescent detection of PAX7 (magenta) and MYOG (green) (G), and corresponding quantifications in 5- (H) and 7-day–injured (I) control and AR^(i)sat-/Y^ mouse TA. DAPI: nuclei. Scale bars, 100 μm (main) and 30 μm (inset). (**J** and **K**) Immunofluorescent detection of MYOG (magenta) and KI67 (green) (J) and corresponding quantification of MYOG/KI67 double-positive cells (K) in 5- and 7-day–injured control and AR^(i)sat-/Y^ mouse TA. White arrows: MYOG/KI67 double-positive cells. DAPI: nuclei. Scale bars, 75 μm (main) and 30 μm (inset). (**L**) H&E staining of control and AR^(i)sat-/Y^ mouse TA, 7 (single injury) and 28 + 7 (double injury) days after injury. Cyan blue arrows: inflammatory cells. Green arrows: interstitial space. Black arrows: central nuclei. Pink arrows: misshaped fibers. Scale bars, 100 μm. (**M**) Immunofluorescent detection of DMD (red) and PAX7 (green) of 28 + 7–day–injured control and AR^(i)sat-/Y^ mouse TA. Scale bars, 50 μm. (**N**) Quantification of PAX7-positive cells in 28 + 7–day–injured control and AR^(i)sat-/Y^ mouse TA. (**O**) Schematic overview of MuSC-autonomous regulation by AR signaling in young muscle. Created in BioRender. D.D. (2026); https://BioRender.com/4q7anrd. Data are mean ± SEM. Statistical tests: [(E) to (F), (H), (I), and (K)] two-way ANOVA with Tukey correction and (N) two-tailed Mann-Whitney test. n.s., not significant; *P < 0.05; **P < 0.01; ***P < 0.001.

Notably, only few genes were differentially expressed in MuSC, suggesting that different cell populations might lay in this cluster (fig. S3F). To further explore the transcriptional changes between control and AR^(i)sat-/Y^ MuSC, we performed a subclustering of MuSC following additional stringent quality control, yielding a final dataset of 445 MuSC (fig. S4, A and B). Unsupervised clustering revealed six distinct MuSC subpopulations (fig. S4B), of which two clusters exhibited an immune signature characterized by the expression of *C1qc*, *Lyz2*, and *Cd68* (fig. S4, B and C). Given recent findings describing these subsets as immunomyoblasts in regenerating muscle (*29*, *31*), we hypothesized that these clusters represent MuSC targeted for immune-mediated clearance. Consequently, we excluded these populations from our analysis and reclustered the remaining four subtypes based on their transcriptional profiles (Fig. 4A; fig. S4, D and E; and table S3).

Our analysis delineated two distinct clusters of quiescent MuSC (clusters 0 and 1), both characterized by the expression of *Pax7* and *Myf5*, while lacking *Myod1* and *Myog* expression (Fig. 4, A and B, and fig. S4E). Moreover, we identified an activated MuSC subcluster (cluster 2), distinguished by the additional expression of *Myod1* and the proliferation marker *Mki67* (Fig. 4, A and B, and fig. S4E). A fully committed myogenic cell population (cluster 3), characterized by *Myog* expression and that of *Mymk* and *Mymx* and originally absent in control individuals, emerged in mutant mice (Fig. 4, A and B, and fig. S4, E and F). In line with this observation, we found that *Ar* expression was confined to quiescent clusters 0 and 1 in control mice, whereas ectopic *Myf5* and *Myod1* expression was noted in cluster 1 in AR^(i)sat-/Y^ individuals (Fig. 4B), indicating that loss of AR in MuSC promotes myogenic commitment. Notably, several genes associated with symmetric division, including *Bmi1*, *Msi2*, and *Zfx*, were up-regulated in mutant mice within cluster 3 (fig. S4G), alongside a broader expression of the fusogenic markers *Mymk* and *Mymx* across a larger subset of cells (fig. S4F), indicative of symmetric differentiation.

To provide evidence of commitment defaults caused by loss of AR in MuSC, we assessed cell cycle distribution within each cluster (Fig. 4C and fig. S4H). Our analysis revealed various perturbations upon AR loss, including cells in G_2_-M in cluster 1 (Fig. 4C). Moreover, in the fully committed cluster 3, the proportion of cells in G_1_ dropped from ∼80% in controls to ∼60% in mutants, whereas those in S phase, absent in control mice, increased by more than 15% in MuSC-AR–deficient mice (Fig. 4C), indicating that they are more prone to mitotic activity.

To investigate the division modalities of MuSC, we performed a series of immunofluorescence analyses. Double staining for PAX7 and KI67 in injured TA muscles from control and AR^(i)sat-/Y^ mice at 5 and 7 dpi revealed a significant reduction in proliferating MuSC in mutant mice (Fig. 4, D to F), indicative of impaired symmetric stem cell divisions. Furthermore, double immunodetection of PAX7 and MYOG at 5 dpi showed a marked increase in PAX7^+^/MYOG^+^ cells, accompanied by a decrease in PAX7^+^/MYOG^−^ cells upon AR loss in MuSC (Fig. 4, G and H). By 7 dpi, both PAX7^+^/MYOG^+^ and PAX7^−^/MYOG^+^ cell populations exhibited a twofold increase in AR^(i)sat-/Y^ mice (Fig. 4, G and I), supporting a shift from symmetric stem cell divisions toward an asymmetric division program.

To determine whether the absence of AR in MuSC alters their mode of division, we performed MYOG and KI67 double immunostaining on injured TA muscles at 5 and 7 dpi. Notably, MYOG^+^/KI67^+^ double-positive cells were detected within regenerating regions of mutant muscles (Fig. 4J), consistent with our scRNA-seq analysis showing an increased proportion of the committed cluster 3, characterized by *Mki67* expression in mutants (fig. S4I). Quantification confirmed that MYOG^+^/KI67^+^ cells were more abundant in AR^(i)sat-/Y^ muscles at both 5 and 7 dpi (Fig. 4K), indicating that MuSC prematurely engage in the differentiation program. To further examine MuSC division behavior, we isolated single myofibers from injured TA muscles at 7 dpi, and analyzed them in vitro to assess the modalities and organization of satellite cell divisions. PAX7, KI67, and MYOG immunostaining revealed abnormalities in AR^(i)sat-/Y^ myofibers. Analysis of division outcomes on isolated fibers from control and AR^(i)sat-/Y^ mice showed the presence of dividing and differentiating MuSC on mutant fibers (fig. S4J). These observations indicate changes in MuSC behavior on isolated fibers and are consistent with altered division modalities under the abovementioned AR-deficient condition. In addition, mutant fibers exhibited nuclear budding-like structures along the fiber surface, a feature consistently absent in control fibers (fig. S4K). Overall, these data are consistent with dysregulated MuSC proliferation and premature engagement of the differentiation program upon loss of AR.

We henceforth hypothesized that increased symmetric differentiation may deplete the stem cell pool, compromising its ability to support effective repair during subsequent regenerative challenges. To test this, we subjected control and AR^(i)sat-/Y^ muscles to a secondary CTX-induced injury 28 days after the primary injury and assessed tissue morphology 7 days later (28 + 7 dpi). At this stage, control muscles exhibited an accelerated regenerative response compared to muscles analyzed at 7 dpi following a single injury (Fig. 4L), consistent with previous reports (*1*, *32*), reflecting a primed regenerative state. This response was characterized by reduced immune cell infiltration (Fig. 4L, cyan arrows), a predominance of centrally nucleated myofibers (Fig. 4L, black arrows), and minimal interstitial matrix deposition (Fig. 4L, green arrows). In contrast, AR^(i)sat-/Y^ muscles displayed exacerbated pathological features, including persistent immune infiltration (Fig. 4L, cyan arrows), multinucleated fibers with pronounced morphological abnormalities, and disorganized fibers with fusion defects and nuclear mislocalization throughout the sarcoplasm (Fig. 4L, pink arrows), together with fat deposition (Fig. 4L, red arrows). Immunolabeling for DMD further confirmed the structural heterogeneity and irregularities in AR^(i)sat-/Y^ muscles at 28 + 7 dpi (Fig. 4M). These defects were accompanied by a threefold reduction in MuSC numbers in AR^(i)sat-/Y^ mice compared to controls, as revealed by quantification of PAX7-positive MuSC at 28 + 7 dpi (Fig. 4N). Overall, these results indicate that AR signaling in MuSC is essential for maintaining the stem cell pool by preventing premature differentiation (Fig. 4O), thereby ensuring effective regenerative capacity during both primary and subsequent injuries.

### AR controls MuSC fate through early chromatin remodeling

**Fig. 5.**
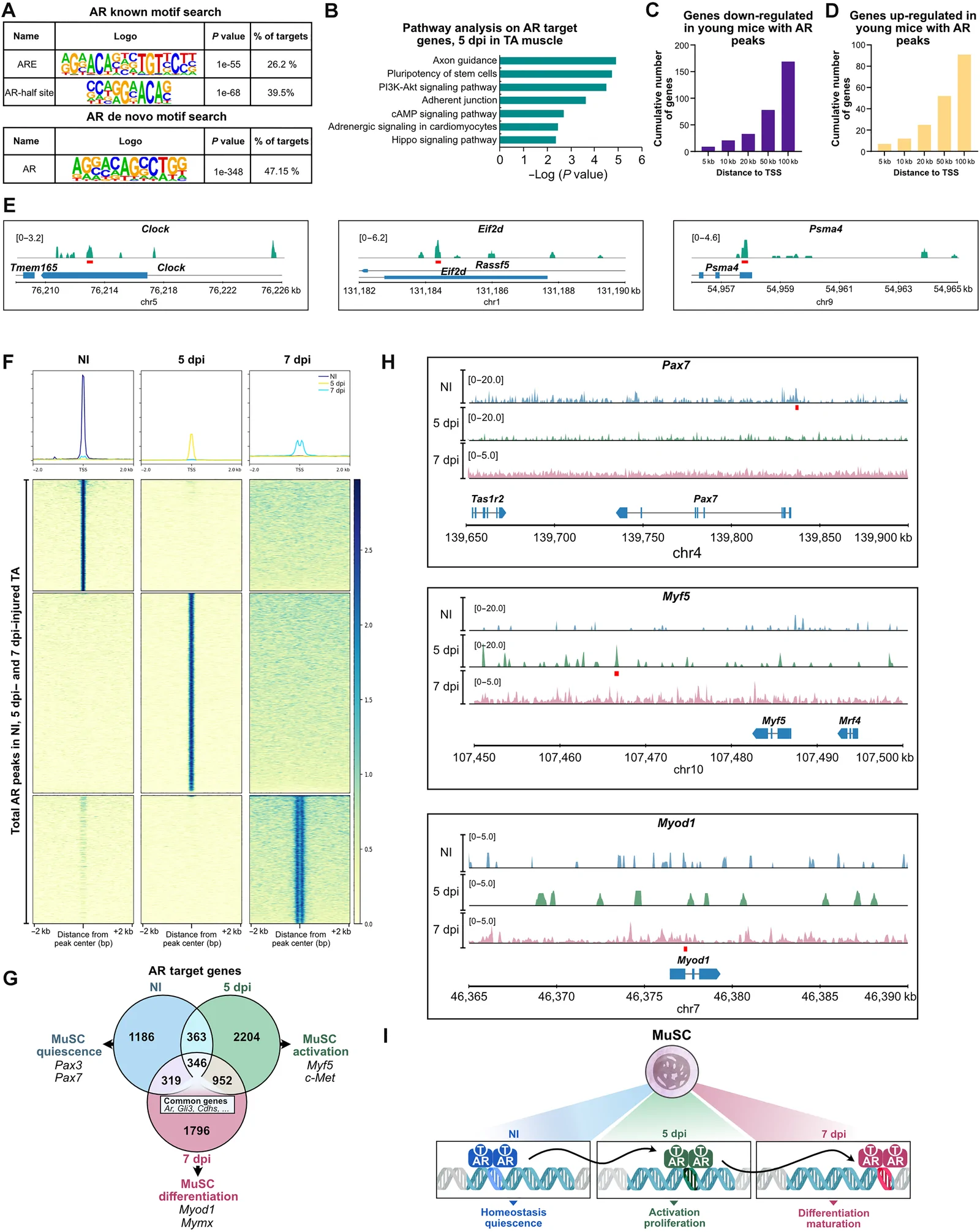
MuSC-AR exhibits time-dependent binding to target genes following injury. (**A**) HOMER known and de novo motif analyses of AR-bound DNA sequences from 5-day–injured TA of young male mice. (**B**) Pathway analysis performed on AR common target genes identified in MuSC of 5-day–injured TA of young male mice. cAMP, adenosine 3′,5′-monophosphate. (**C** and **D**) Cumulative number of down-regulated genes (C) and up-regulated genes (D) in MuSC of 5-day–injured TA of young male mice, with an AR peak located at the indicated distances from their TSS. (**E**) AR localization at the *Clock*, *Eif2d*, and *Psma4* loci. Binding sites are indicated in red. (**F**) Average tag density profiles and corresponding tag density map of AR in NI, 5-, and 7-day–injured TA muscles of young male mice, +/−2 kb from the AR peak center. (**G**) Overlap among genes bound by AR, in NI, 5-, and 7-day–injured TA muscles of young male mice. (**H**) AR localization in MuSC in NI, 5-, and 7-day–injured TA muscles of young male mice, at indicated loci. (**I**) Schematic overview of AR-mediated temporal regulation and chromatin binding dynamics in MuSC during regeneration at young age. AR displays stage-specific chromatin occupancy throughout muscle regeneration. In uninjured muscle, MuSC-AR supports homeostasis. Following injury, AR translocates to genomic regions that drive regenerative programs, favoring genes involved in MuSC activation and proliferation at 5 dpi and shifting toward loci promoting differentiation and maturation by 7 dpi. Created in BioRender. D.D. (2026); https://BioRender.com/4q7anrd.

To capture early AR-dependent chromatin events with the potential to drive the scRNA-seq-detected transcriptomic alterations and downstream regenerative defects, we performed CUT&RUN profiling of AR and the active chromatin mark H3K4me2 in FACS-isolated MuSC of young mice at 5 dpi. Consistent with our observations at 7 dpi, CUT&RUN analysis at 5 dpi identified 7612 AR binding peaks, primarily localized to intronic and intergenic regions (fig. S5A), associated with 7040 genes. Most of these AR-bound loci coincided with H3K4me2 enrichment at promoter regions (fig. S5, B and C). Motif analysis using HOMER revealed that 50.6% of AR peaks contained canonical AR binding motifs (Fig. 5A), confirming the specificity of AR-DNA interactions. Functional enrichment analysis of AR target genes demonstrated significant involvement in pathways critical for stem cell homeostasis and tissue remodeling, notably the HIPPO and PI3K-Akt signaling pathways (Fig. 5B). Similar to what we described at 7 dpi, gene-centric analysis revealed that AR is mainly recruited within a 100-kb window of more than 50% of DEGs (Fig. 5, C and D). Representative AR binding loci at key genes involved in these regulatory pathways, including *Clock*, *Eifd2*, and *Psma4*, are depicted in Fig. 5E. Together, these findings demonstrate that AR engages early with the MuSC chromatin landscape, preferentially at distal regulatory elements enriched in active histone marks, to control the transcriptional programs governing stem cell fate and tissue remodeling.

### AR dynamically repositions upon injury to govern time-dependent regenerative genomic programs

The transcriptomic discrepancies observed between 5 and 7 dpi led us to question AR positioning on the DNA during muscle repair. deepTools analysis demonstrated minimal overlap and distinct peak profiles for each state between AR-bound chromatin landscapes 0 (basal), 5, and 7 dpi (Fig. 5, F to H). This temporal specificity was further confirmed by comparative analysis of the genes bound by AR in those specific clusters (Fig. 5F). The genes commonly controlled by AR during the regeneration process were mainly implicated in cell-cell adhesion or in the connection with the synapse, essentially via cadherins (e.g., *Cdh2*, *Cdh6*, and *Cdh7*) and included *Ar* itself and *Gli3* that is essential for MuSC injury memory (Fig. 5G) (*33*). In the absence of injury, AR binding was enriched at genomic loci associated with MuSC stemness, including *Pax3*, *Pax7*, and *Kdm1a* (*Lsd1*) (Fig. 5, G and H), while AR-bound landscape profile markedly shifted at 5 dpi, targeting genes implicated in MuSC activation, such as *Myf5* and *Tead1*, and asymmetric division such as *Aurkb* or muscle commitment including *Myh4* and *Myh9* (Fig. 5, G and H). Seven dpi, AR was further reallocated to loci related to myogenic differentiation such as *Myod1*, *Mef2a*, and *Mef2d* and asymmetric division such as *Numb* and *Pard3* (Fig. 5, G and H). Representative genes exhibiting AR binding loci in a temporal and stage-specific manner during regeneration such as *Pax7*, *Myf5*, and *Myod1* are depicted in Fig. 5H. Collectively, these findings reveal that AR is dynamically repositioned in MuSC following injury, engaging stage-specific genomic loci to orchestrate transcriptional programs that govern activation and lineage commitment via MRFs (Fig. 5I). This temporal chromatin remodeling ensures a balanced stem-to-committed transition, safeguarding the MuSC pool for future regenerative demands in young adults.

### Age-related regenerative decline in skeletal muscle is causally linked to diminished androgen signaling

**Fig. 6.**
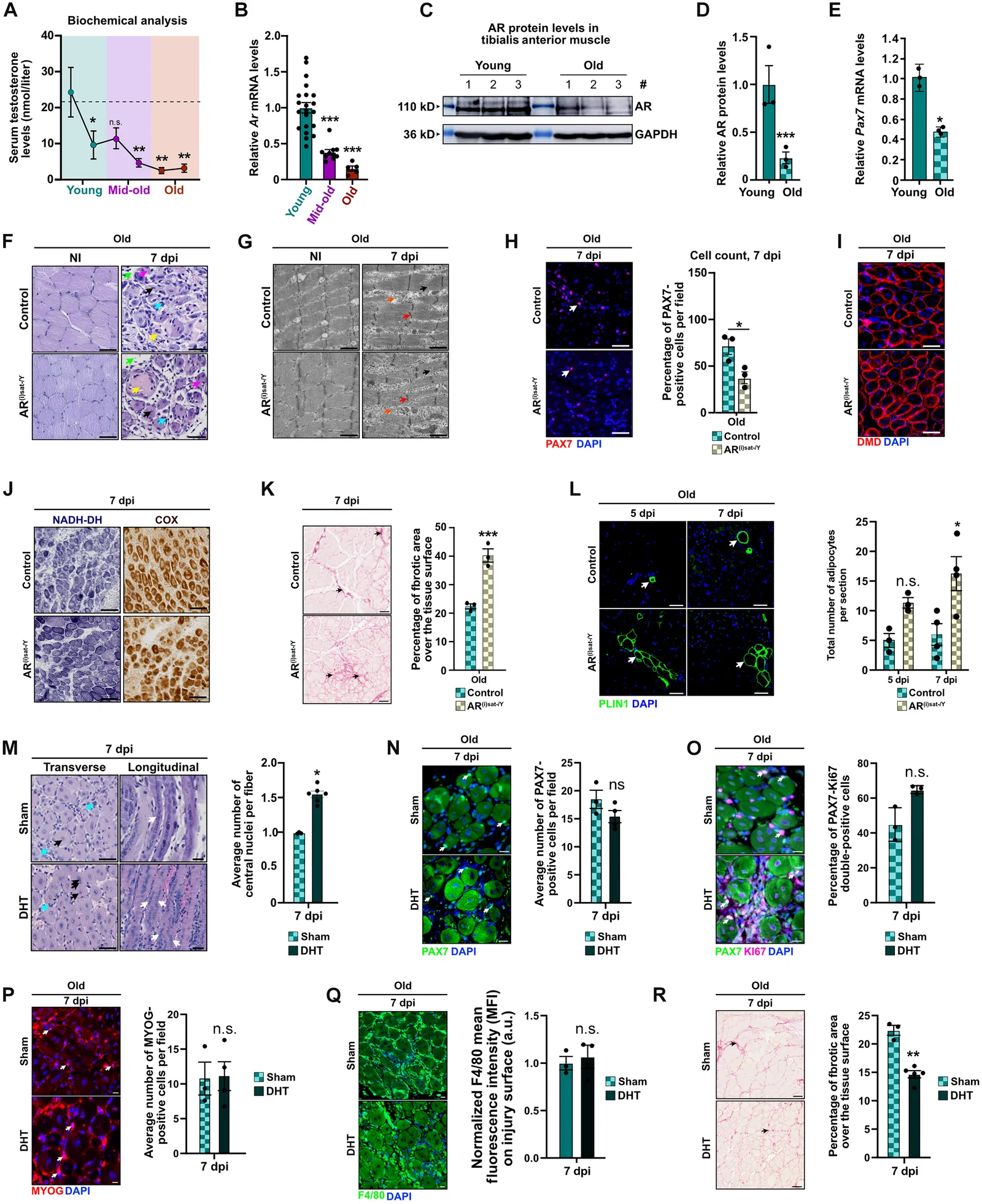
Age-related androgen decline alters skeletal muscle repair machinery. (**A**) Mouse serum testosterone levels. Dashed line: basal levels. (**B**) Relative *Ar* transcript levels in mouse TA. (**C** and **D**) Western blot analysis (C), and corresponding quantification of indicated proteins (D) in mouse TA. Glyceraldehyde-3-phosphate dehydrogenase (GAPDH) was used as a loading control. (**E**) Relative *Pax7* transcript levels in mouse TA. (**F**) H&E staining of NI and 7 dpi control and AR^(i)sat-/Y^ mouse TA. Arrows: black, centrally nucleated fibers; blue, inflammation; yellow, necrosis; green, interstitial space; pink, misshaped myofibers; orange, fibrosis. (**G**) Ultrastructure of NI and 7-day–injured control and AR^(i)sat-/Y^ mouse TA. Arrows: black, M-line; red; Z-line; pink, T-tubules misalignments; orange, fibrosis; green, A-band; yellow, I-band. (**H**) PAX7 (red) immunofluorescence, and corresponding quantification in 7-day–injured control and AR^(i)sat-/Y^ mouse TA. White arrows: PAX7-positive cells. DAPI: nuclei. (**I**) DMD (red) immunofluorescence of 7-day–injured control and AR^(i)sat-/Y^ mouse TA. DAPI: nuclei. (**J**) NADH-DH and COX activity staining in 7-day–injured control and AR^(i)sat-/Y^ mouse TA. (**K**) Sirius Red and corresponding quantification in 7-day–injured control and AR^(i)sat-/Y^ mouse TA. Black arrows: collagen fibers. (**L**) PLIN1 (green) immunofluorescence and corresponding adipocyte quantification in 5- and 7-day–injured control and AR^(i)sat-/Y^ mouse TA. White arrows: adipocytes. DAPI: nuclei. (**M**) H&E staining and corresponding quantification of centronuclei average in TA of sham-operated or 5-α-dihydrotestosterone (DHT)–supplemented mice, 7 dpi. Arrows: cyan, inflammation; black, centronuclei; white, nuclei alignments. (**N** to **Q**) PAX7 (green) (N), PAX7 (green) and KI67 (magenta) (O), MYOG (red) (P), and F4/80 (green) (Q) immunofluorescence and corresponding quantifications in sham-operated or DHT-supplemented old mouse TA, 7 dpi. DAPI: nuclei. a.u., arbitrary units. Scale bars, 75 μm [(F) and (H)], 1 μm (G), 100 μm [(I) and (L)], 200 μm (J), 250 μm (K), 150 μm [(M) transverse], 100 μm (longitudinal), 50 μm [(N) and (Q)], 20 μm [(O) and (P)]. Data are mean ± SEM. Statistical tests: [(A) and (B)] ordinary one-way ANOVA, (L) two-way ANOVA with Tukey’s correction, and [(D), (E), (H), (K), and (M) to (Q)] two-tailed Mann-Whitney. n.s., not significant; *P < 0.05; **P < 0.01; ***P < 0.001.

Given the age-related decline in MuSC number and regenerative potential, we hypothesized that diminished androgen signaling may underlie this process. We thus quantified serum testosterone levels in mice at different ages and observed a progressive decrease from ∼25 nmol/liter in young mice to a plateau of ∼5 nmol/liter in old ones (Fig. 6A). This was accompanied by a corresponding decrease in *Ar* expression at both transcript and protein levels in noninjured (NI) TA muscles (Fig. 6, B to D), as well as a decrease in the number of MuSC expressing AR, compared to young mice (fig. S6A). Notably, the transcript levels of the MuSC marker *Pax7* were diminished in NI TA of old control mice (Fig. 6E).

To determine the functional consequences of these baseline deficits, we induced CTX injury in 1-year-old (hereafter old) control and mutant mice, treated with TAM at 9 weeks and subsequently aged under natural conditions (fig. S6B). As expected, and consistent with previous reports (*10*, *11*), old male mice exhibited markedly impaired regenerative capacity compared to young individuals (fig. S6C and S1K). To dissect the additional contribution of MuSC-specific AR loss to this decline, we directly compared aged mutants to age-matched controls. Notably, while PAX7-positive MuSC in control mice exhibited faint AR expression, it was entirely absent in AR^(i)sat-/Y^ mutants (fig. S6B and S6D). At 7 dpi, aged AR^(i)sat-/Y^ muscles displayed increased interstitial space, sustained immune infiltration, and misshaped regenerating fibers when compared to controls (Fig. 6F). Ultrastructural analysis confirmed these impairments, showing disrupted sarcomere organization, fragmented Z-lines, and increased ECM deposition (Fig. 6G). These structural impairments were accompanied by a significant decrease in MuSC numbers (Fig. 6H), and deformation of regenerating fibers (Fig. 6I and S6E). In addition, recovery in aged muscles was accompanied by a pronounced shift toward oxidative metabolism (Fig. 6J), increased fibrosis (Fig. 6K) and adipocyte emergence (Fig. 6L), highlighting the pivotal role of AR signaling in safeguarding the structural and functional fidelity of regenerating muscle. Notably, the regenerative phenotype observed in aged control muscles partially recapitulated features seen in young AR^(i)sat-/Y^ mutants, whereas the combination of aging and MuSC-specific AR loss further exacerbated these defects, highlighting the additive impact of hormonal decline and receptor deficiency on regenerative capacity (fig. S6C).

To determine whether the regenerative impairment is causally linked to androgenic insufficiency, we tested whether restoring androgen signaling could reverse aspects of the aged phenotype. We thereby supplemented old mice with 5-α-dihydrotestosterone (DHT) using implants. DHT supplementation in old mice led to increased AR protein levels in gastrocnemius skeletal muscle 1 week after implantation (fig. S6F). At 7 dpi, DHT-treated mice exhibited significantly improved muscle regeneration, as evidenced by enhanced myofiber morphology and an increased number of nuclei per regenerating fiber (Fig. 6M). Although the total number of MuSC was not significantly altered (Fig. 6N), we observed a slight increase in proliferating PAX7/KI67 double-positive cells (Fig. 6O), which likely contributes to the increased number of centrally located nuclei and enhanced myogenic fusion. No significant differences were detected in differentiation capacity or immune infiltration, as assessed by MYOG and F4/80 staining, respectively (Fig. 6, P and Q). In addition, fibrosis analysis revealed a marked reduction in fibrotic area, with decreased collagen deposition within the ECM (Fig. 6R). Together, these findings provide direct evidence that age-related androgen deficiency causally contributes to impaired aging skeletal muscle regeneration.

### Androgen signaling orchestrates distinct gene networks in young and old individuals leading to similar phenotypes

**Fig. 7.**
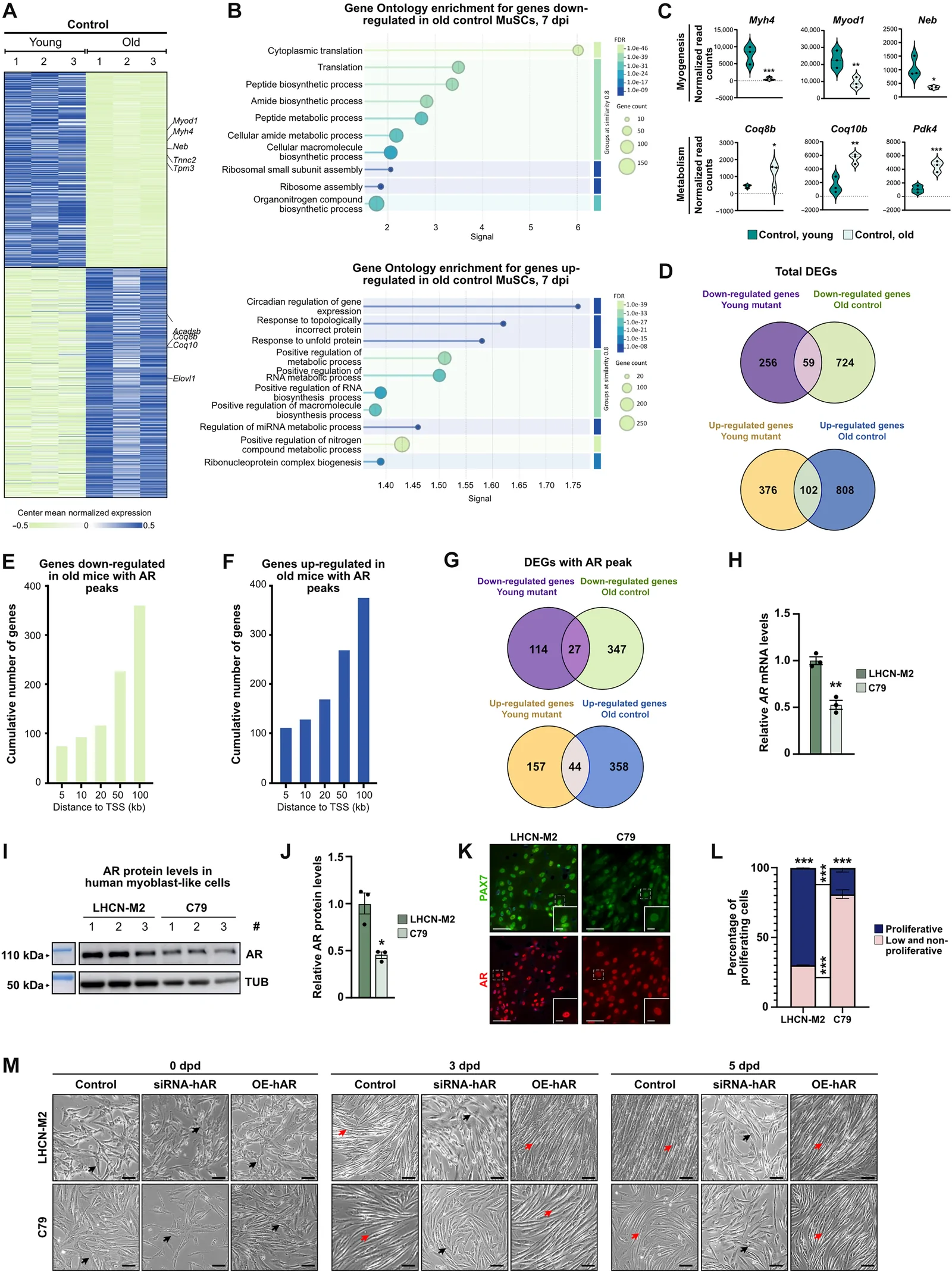
AR impact on MuSC function in young and aged muscle. (**A** to **C**) Heatmap depicting the mean centered normalized expression of down- and up-regulated genes from SMART-seq analysis performed on MuSC from 7-day–injured TA of old and young mice (A), and corresponding Gene Ontology enrichment (B) and violin plots (C). (**D**) Venn diagrams showing intersection among DEGs in MuSC of control old mice and AR^(i)sat-/Y^ young mice. (**E** and **F**) Cumulative number of down- (E) and up-regulated genes (F) in MuSC of 7-day–injured TA of old mice, with AR peak located at indicated distances from TSS. (**G**) Venn diagrams showing intersected genes with AR peaks among DEGs in MuSC of control old mice and AR^(i)sat-/Y^ young mice. (**H**) Relative *AR* transcript levels in LHCN-M2 and C79 myoblasts. (**I** and **J**) Representative AR Western blot analysis (I), and corresponding quantification (J) in control LHCN-M2 and C79 myoblasts. α-TUBULIN (TUB) was used as a loading control. (**K**) Representative immunofluorescent detection of PAX7 (in green) and AR (in red) in LHCN-M2 and C79 cells. Scale bars, 100 μm (main) and 10 μm (inset). (**L**) Quantification of the percentage of high- and low/nonproliferative control LHCN-M2 and C79 myoblasts determined by CellTrace analysis. (**M**) Representative bright-field images of control (CTR), AR–small interfering RNA (siRNA) transfected (siRNA-hAR), and AR-plasmid transfected (OE-hAR) LHCN-M2 and C79 cells, 0, 3, and 5 days postdifferentiation (dpd). Black arrows show nondifferentiated cells. Red arrows label differentiated cells. Scale bars, 100 μm. Data are mean ± SEM. Statistical tests: (C) Wald test, [(H) and (J)] two-tailed Mann-Whitney test, and (L) two-way ANOVA with Tukey correction. *P < 0.05; **P < 0.01; ***P < 0.001.

To understand how the age-related decline in androgen signaling affects MuSC regenerative programs, we first compared the transcriptomic profiles of old control MuSC to those of young counterparts at 7 dpi. This analysis identified 1709 DEGs, including 798 down-regulated and 911 up-regulated transcripts (Fig. 7A and fig. S7A). Pathway enrichment analyses of the down-regulated gene set highlighted a significant suppression of pathways related to protein synthesis and peptide/amide metabolism (Fig. 7B), suggesting a reduced biosynthetic capacity in aging MuSC. Notably, several genes essential for myogenic differentiation and structural regeneration were significantly down-regulated. These included *Myh4* and *Myod1* in addition to genes involved in sarcomere organization and contractile function, such as nebulin (*Neb*), *Tpm3*, and troponin C type 2 (*Tnnc2*) (Fig. 7, A and C). This transcriptional repression is consistent with the defective regenerative phenotype and histological and ultrastructural impairments observed in aged control mice at 7 dpi. Conversely, pathway analyses on the 911 genes up-regulated in MuSC from old control mice compared to young control counterparts revealed that these genes were predominantly associated with metabolic and biosynthetic processes, notably the regulation of RNA, microRNA, nitrogen metabolism, and nucleobase-containing compound metabolic pathways (Fig. 7, A and B) but also oxidative metabolism processes (e.g., *Acadsb*, *Coq8b*, *Coq10b*, and *Pdk4*) (Fig. 7, A and C), collectively indicating a metabolic transition toward oxidative phosphorylation in old MuSC. These molecular findings align with the histological evidence of enhanced oxidative metabolism observed at 7 dpi in old individuals.

Because aged control muscles exhibit histological and structural features reminiscent of young AR^(i)sat-/Y^ ones, we next examined age-regulated genes to determine whether these shared changes involve AR-dependent regulatory programs. To this end, we compared age-regulated genes with those deregulated in young mutants and notably identified only 59 down-regulated and 102 up-regulated genes common between young and aged MuSC (Fig. 7D). A substantial fraction of DEGs specific to aged MuSC exhibited AR binding within 100 kb of their TSSs (Fig. 7, E and F), indicating potential direct AR regulation. To assess the extent to which age-associated transcriptional changes mimic AR loss in youth, we compared AR direct targets identified in young mutant MuSC with age-regulated ones in old controls. This analysis revealed only limited overlap with AR-bound genes (Fig. 7G), indicating that the molecular mechanisms underlying AR regulation in young MuSC differ from those driving age-associated transcriptional changes, although Gene Ontology analyses revealed similar functional enrichment (fig. S7, D and F). This limited convergence highlights that, while AR deficiency in MuSC at young age partially recapitulates transcriptional features of aging, the molecular underpinnings differ, reflecting the distinct role of AR during development as opposed to the combined decline of hormone and receptor signaling observed in the elderly. Together, these findings support a model in which AR orchestrates regenerative transcriptional programs in MuSC via enhancer-driven mechanisms, a regulatory framework that persists with age but becomes functionally altered.

To extend our findings to a human model, we determined AR expression in LHCN-M2 and C79 myoblasts, isolated from young and aged male individuals, respectively. Notably, AR expression was reduced by approximately 50% in C79 compared to LHCN-M2 myoblasts at both the transcript (Fig. 7H) and protein levels (Fig. 7, I and J), in addition to the PAX7 levels (Fig. 7K). Moreover, LHCN-M2 cells displayed a higher propensity for proliferation (Fig. 7L and fig. S7I) and differentiation compared to C79 cells (Fig. 7M), mirroring the differences observed between young and aged mice. In agreement, AR knockdown in both cell lines strongly impaired differentiation, as indicated by the conservation of a fibroblast-like morphology and lack of the characteristic spindle-shaped structure observed in control cells, 5 days postdifferentiation (dpd) (Fig. 7M and fig. S7, J and K). Conversely, while AR overexpression did not have flagrant effects on LHCN-M2 cell differentiation, a premature more efficient myogenesis was observed in C79 cells, in the presence of elevated AR levels (Fig. 7M and fig. S7, L to N). Collectively, these findings demonstrate that intact AR signaling, requiring both sufficient ligand and receptor, is essential for efficient myoblast differentiation and, by extension, effective skeletal muscle regeneration.

## DISCUSSION

Maintenance of the MuSC reservoir requires mechanisms that preserve quiescence while ensuring that repair proceeds with structural and functional fidelity. In young male skeletal muscle, AR expression concentrates in the most quiescent, self-renewing MuSC states and is largely extinguished as cells commit. Conditional loss of AR in MuSC disrupts dormancy, accelerates cell-cycle entry, and drives symmetric differentiation on the expense of stem cells renewal, leading to attrition of the regenerative pool. Notably, bulk RNA-seq at 7 dpi reveals significant impairments in *Pax7*, *Myf5*, *Myod1*, *Myog*, and *Mymx* expression in AR^(i)sat-/Y^ cells, whereas scRNA-seq at 5 dpi does not detect these changes. This discrepancy reflects differences in both regeneration stage and analytical resolution as bulk RNA-seq captures population-wide transcriptional shifts later in repair, whereas scRNA-seq interrogates cell-state–specific signatures at an earlier time point without implying a biological inconsistency. On this basis, we here identify AR as lineage-intrinsic indicator of MuSC stemness in males, functionally distinct from its canonical anabolic role in myofibers (*16*, *25*, *26*, *34*).

This AR-stemness relationship fits within a broader endocrine framework across the male life course. At young age, androgens promote the establishment of a long-lived quiescent MuSC pool from juvenile precursors through a Notch-dependent pathway (*18*). With aging, declining systemic androgens correlate with reduced MuSC number and impaired repair (*35*), and androgen supplementation restores regenerative capacity in low-androgen settings (*36*, *37*). Consistent with this trajectory, our MuSC-specific AR knockout phenotypically recapitulates features of aging-associated stem cell exhaustion, including reduced responsiveness to repeated injuries, suggesting that androgen decline and diminished AR activity converge on shared mechanisms that erode the MuSC reservoir. However, transcriptomic differences observed between AR-deficient young MuSC and those from aged controls may also reflect distinct underlying causes, namely, the selective inactivation of AR in otherwise intact young MuSC versus the broader loss of stem cells with age, along with age-related alterations in the muscle microenvironment (*38*). These observations indicate that androgen decline and AR loss exert distinct yet complementary effects; the former reduces stem cell abundance and responsiveness, while the latter disrupts intrinsic programs of quiescence and fate. In aged AR-deficient mutants, these deficits appear to sum, producing an exacerbated phenotype that underscores the dual contribution of hormone and receptor to MuSC maintenance.

We also observe that AR expression in young individuals persists for weeks after injury in MuSC derived from self-renewal, reflecting early symmetric divisions driven by AR that maintain the quiescent stem pool for future regenerative demands. In contrast, differentiating MuSC gradually lose AR, showing that AR not only supports stem cell expansion but also controls asymmetric divisions to produce committed cells for repair. Without AR, MuSC divisions favor committed daughters over self-renewal, depleting the reserve and impairing regeneration after repeated injury.

The proliferation defects observed at 5 to 7 dpi may not solely reflect impaired proliferative capacity. They could also arise from alterations occurring during the earliest phases of regeneration, including differences in the timing of quiescence exit, initial MuSC activation, or early cell survival within the first hours after injury. As these events were not examined in the present study, their contribution cannot be excluded and warrants further investigation.

The AR-dependent balance between renewal and differentiation in male MuSC has not been shown in vivo before. Earlier studies using female-derived C2C12 myoblasts showed that DHT enhances proliferation, but these cells lack true quiescence and do not represent adult male MuSC (*20*, *39*, *40*). In contrast, our findings reveal that physiological androgen levels via AR in male MuSC dynamically regulate their fate during regeneration.

MuSC-AR influences more than lineage fate in skeletal muscle. It promotes proliferation and differentiation in myogenic cells (*41*, *42*), and its loss in our mouse model leads to fusion defects, fiber orientation defaults, and impaired ultrastructure, reflecting not only reduced progenitor output but also a disrupted regenerative program in these progenitors. This contradicts earlier claims that MuSC-AR is dispensable for regeneration (*23*–*25*), likely because their model did not disrupt AR in the early, true stem MuSC population. Our data establish MuSC-intrinsic AR as a crucial regulator of androgen-driven muscle repair in males. This signaling axis also shapes metabolic identity. While myofiber-AR supports glycolytic metabolism, its absence in MuSC at young age shifts regenerated fibers toward oxidative pathways, which was recapitulated in aged individuals. Seahorse assays and histology confirm this shift, consistent with findings in AR-deficient myofibers (*25*, *43*), AR-related disorders (*44*), and aging muscle (*45*, *46*). Notably, myofiber-AR loss is linked to increased β-oxidation gene expression and reduced complex IV components (*25*, *47*), although this latter feature was absent in our model, possibly due to reactive oxygen species accumulation impairing electron transport and causing mitochondrial stress (*48*).

At the tissue level, MuSC-AR deletion in young individuals reveals increased inflammation, fibrosis, and adipocyte accumulation at the site of injury. Similar disturbances occur in androgen-deprived states, where impaired inflammatory resolution and accelerated fibro-adipogenic drift are observed (*49*, *50*). These findings show that MuSC are not passive recipients but active transmitters of androgen-sensitive signals shaping their microenvironment. This supports a bidirectional model in which AR in MuSC governs intrinsic fate decisions while orchestrating stromal and immune cross-talk during muscle repair. While our data indicate that MuSC-AR influences macrophages and FAPs, these effects are likely indirect. CellChat analysis did not reveal significant differences in secreted signaling molecules, and in vitro coculture experiments were precluded by the poor adherence of FACS-isolated AR^(i)sat-/Y^ MuSC. Therefore, MuSC-AR appears to shape the regenerative niche predominantly through indirect intercellular mechanisms. In AR-deficient muscles, macrophages prematurely express anti-inflammatory markers, yet the total inflammatory burden persists longer, reflecting an uncoupling between phenotypic transition and effective clearance. This aberrant trajectory likely contributes to impaired regeneration and highlights the nuanced role of MuSC-AR in coordinating the immune niche. Our CellChat analysis further suggests that AR-deficient MuSC perturb intercellular communication with macrophages and FAPs, although the precise signaling mediators remain to be defined. Identifying these factors represents a compelling avenue for future work, as it would illuminate the mechanisms by which MuSC orchestrate niche remodeling and coordinate regeneration.

To coordinate these diverse, stage-specific functions during muscle regeneration, we propose a dynamic chromatin occupancy model in which AR sequentially engages distinct transcriptional programs as MuSC progress from quiescence through activation to commitment. These nuclear receptor mobility and partner switching are well documented in other systems (*51*–*54*). Emerging evidence in skeletal muscle shows that AR collaborates with SMAD signaling to enhance bone morphogenetic protein–driven transcriptional programs that regulate muscle mass and quality (*55*). In aging and pathological expansions of the AR-polyglutamine tract, altered chromatin binding and disrupted cofactor interactions have been observed (*56*, *57*), suggesting that reduced AR availability with age may limit its genomic sampling and assembly of stage-specific complexes necessary to maintain stemness and sequentially enable differentiation.

In sum, MuSC-AR preserves quiescence, sustains the stem cell pool, shapes glycolytic identity, and orchestrates niche remodeling. Its decline leads to reserve loss, metabolic drift, and regenerative failure (fig. S7O), highlighting MuSC-AR as a therapeutic target in aging and disease.

## MATERIALS AND METHODS

### Mouse studies

#### Housing

Mice were maintained on a C57BL/6 background and housed in a temperature- and humidity-controlled facility under a 12-hour light/dark cycle, with ad libitum access to standard rodent chow (2800 kcal/kg; Usine d’Alimentation Rationnelle, Villemoisson-sur-Orge, France) and water. Breeding and maintenance followed institutional guidelines, and all experiments were conducted in an accredited facility in compliance with French and EU regulations on laboratory animal use (Directive 2010/63/EU). Procedures were approved under the number 48089-2024022308449411 by the local ethical committee (Comité éthique en experimentation animale–CEEA17, Strasbourg, France) and by the French Ministry of Research (MESR). Mice were euthanized via cervical dislocation, and muscles were harvested for histological and molecular analysis.

#### Generation of AR^(i)sat−/Y^ mutant mice

For satellite cell–specific AR ablation, AR^L2/L2^ female mice, carrying LoxP sites flanking chromosome X exon 1 encoding the AR N-terminal domain (*58*), were intercrossed with *Pax7*-CreER^T2^ male mice (*59*) to generate *Pax7*-CreER^T2^/AR^L2/Y^ mice, which express CreER^T2^ recombinase selectively in satellite cells. To induce recombination, 9-week-old AR^L2/Y^ control males and their *Pax7*-CreER^T2^/AR^L2/Y^ premutant littermates received intraperitoneal TAM injections (1 mg per mouse per day) for five consecutive days, generating control and AR^(i)sat−/Y^ mutant mice. Alternatively, mice were intercrossed with a *Rosa26*-YFP reporter line (*60*) to generate *Pax7*-CreER^T2^/AR^L2/Y^/*Rosa26*-YFP^Tg/0^ mice with one YFP transgene copy, allowing selective labeling of satellite cells and lineage tracing of their progeny. *Pax7*-CreER^T2^/*Rosa26*-YFP^Tg/0^ mice lacking the floxed *Ar* allele were used as additional controls. Mice were analyzed at 3 months of age (young adults) or at 1 year of age (aged cohort). Primer sequences required for genotyping polymerase chain reaction (PCR) are listed in table S4.

#### Mouse treatments

For CTX-induced muscle injury model, 3-month young adults or at 1-year aged mice were anesthetized with 2.5% isoflurane using a SomnoSuite device. To induce muscle damage, 20 μl of 25 μM CTX (Latoxan, Valence, France, L8102), dissolved in phosphate-buffered saline (PBS), was injected into the TA muscle.

For androgen supplementation, DHT implants were inserted subcutaneously in the intrascapular region of 21-month-old wild-type male mice, as described (*61*). Muscle injury was induced by CTX injection 3 weeks postimplantation to avoid off-target effects caused by DHT burst.

### Testosterone serum level measurement

Blood was collected from mice in 50 mM EDTA tubes (Sigma-Aldrich), and plasma was isolated by centrifugation at 3000*g* for 15 min at 4°C. The supernatant was collected, and serum testosterone levels were quantified using the Testosterone Rat/Mouse ELISA Kit (LDN, AR E-8000R), following the manufacturer’s instructions.

### Contractile measurements

In situ isometric TA muscle force in response to nerve stimulation was measured as described (*26*). Briefly, specific maximal tetanic force (in millinewtons per square millimeter) was determined by dividing the measured force by the estimated CSA of the muscle. Assuming a cylindrical muscle shape with a density of 1.06 mg/mm^3^, the CSA was calculated as the volume of the muscle divided by its length. The optimal muscle length (*L*_0_) was defined as the length at which maximal tetanic force was observed. All data collected from the muscle lever system were recorded and analyzed using a microcomputer with the PowerLab/4SP system (AD Instruments).

### Histological analyses

#### Muscle histology

Muscles were either snap frozen in liquid nitrogen–cooled isopentane for cryosections or fixed in 4% paraformaldehyde (PFA) overnight at 4°C and then washed in PBS and 70% ethanol and embedded in paraffin. Cryosections (10 μm thick) were prepared at −20°C using a 2800 Frigocut cryostat (Leica, St. Jouarre, France) and kept at −20°C until further analysis. Paraffin sections (5 μm thick) were cut at room temperature (RT) using a microtome (Leica RM, 2145, 0737/09.1998) and stored at 4°C until further analysis. Hematoxylin and eosin, and Masson’s trichrome staining were performed on paraffin sections, while NADH-DH and COX activity staining were assessed on cryosections as described (*25*, *60*, *62*).

#### Quantification of collagen deposition

Collagen content assessed by Sirius Red staining (Picro-Sirius Red Stain, Pint, StatLab, STPSRPT) was imaged under a bright-field microscope or under polarized light using the Axioscan 7 (Carl Zeiss) slide scanner. Birefringent collagen fibers were visualized on the basis of their differential light refraction properties, allowing for the selective detection of collagen deposition. Quantification was performed by analyzing the polarized signal intensity and distribution across the tissue using standardized image analysis parameters established by the in-house anatomic pathology platform. Custom macro for CSA measurements and Sirius Red quantification are available on GitLab and GitHub, respectively (CSA: the BIOP Cellpose extension guide; Sirius Red: https://github.com/Hugues-Jacobs/Fibrosis_analysis).

#### Immunofluorescence staining

Paraffin and cryosections were processed using distinct protocols tailored to antibody requirements. Paraffin sections underwent deparaffinization, and antigen retrieval was done under controlled temperature and pressure conditions for 20 min in either tris-hydroxymethyl-aminomethane (tris buffer) (pH = 9.0) or in SignalStain Citrate Unmasking Solution (Cell Signaling Technology, 14746S). In contrast, frozen sections were fixed for 10 min in 4% PFA at RT, and permeabilized with PBST for 15 min at RT. Afterward, paraffin or frozen sections were incubated overnight at 4°C with the primary antibodies diluted in PBST directed against AR (rabbit, Abcam, ab108341; 1:500), PAX7 [rabbit, Thermo Fisher Scientific, PA1-117 (1:400); or mouse, DSHB, AB_528428 (1:50)], MYOG (Abcam, ab1835; 1:400), ACTN1 (mouse, Abcam, ab9465; 1:400), DMD [mouse, IGBMC, 1Dystro1D2, (1:1000); or rabbit, Sigma-Aldrich, D8168 (1:400)], KI67 (rat, R&D Systems, 14-5698-82; 1:500), LAMA2 (rat, Sigma-Aldrich, L0663-.1ML; 1:500), eMyHC (mouse, DSHB, F1.652; 1:20), F4/80 (rabbit, Cell Signaling Technology, 70076; 1:400), and PLIN1 (rabbit, Abcam, Ab3526; 1:200). Mouse (Santa Cruz Biotechnology, SC-2025; 1:500), rat (Vector Laboratories, I-4000-1; 1:500), or rabbit (PeproTech, 500-P000; 1:500) immunoglobulins G (IgGs) were used as controls. Sections were rinsed with wash buffer and incubated with appropriate Alexa-Fluor–conjugated secondary antibodies with different fluorescent wavelengths (488, 555, or 647 nm; 1:500; Invitrogen: a32732, a32723, a21206, a21424, a48265, a32733, and a32728) in permeabilization buffer for 45 min at RT. After wash, slides were mounted with Fluoromount Aqueous Mounting medium containing 4′,6-diamidino-2-phenylindole (DAPI; Invitrogen, 00-4959-52).

#### Fiber CSA measurements

Muscle cross sections were stained with anti-DMD antibody (Abcam, ab15277; 1:500) to mark the sarcolemma surrounding each fiber. CSAs were quantified using QuPath (open-source image analysis software) integrated with a customized Groovy macro (available via GitLab). Briefly, individual fibers were identified on the basis of the intensity and continuity of the stained sarcolemma surrounding each fiber by segmentation. Areas were measured after background subtraction and automated thresholding.

#### Quantification of F4/80 and CD163 MFI

Fluorescence images of injured TA muscles immunostained for F4/80 and CD163 were analyzed using the QuPath software (QuPath-0.5.0). Regions of interest (ROIs) corresponding to the injured areas were annotated manually, and a pixel classifier was trained on these annotations to categorize pixels as background/negative, positive signal, or other nonpositive signals. Fluorescence intensity thresholds were set manually for each marker to ensure accurate detection of positive staining. MFI within the annotated ROIs was then measured and exported for further analysis.

#### Muscle ultrastructural analysis

TA muscle samples (1 mm^2^) were fixed overnight at 4°C by immersion in a mix of 2.5% glutaraldehyde and 2.5% paraformaldehyde diluted in 0.1 M cacodylate buffer (pH 7.4), washed in the same buffer for 30 min, and stored at 4°C. Postfixation was performed with 1% osmium tetroxide in 0.1 M cacodylate buffer for 1 hour at 4°C, followed by dehydration through a graded ethanol series (50, 70, 90, and 100%) and propylene oxide (30 min each). Samples were oriented longitudinally or transversally and embedded in Epon 812. Ultrathin sections (70 nm) were obtained and contrasted with uranyl acetate and lead citrate. Imaging was performed at 70 kV using a Morgagni 268D electron microscope, with digital acquisition via a MegaView III camera (Soft Imaging System).

### Cell culture analyses

#### Cell culture and treatments

LHCN-M2 and C79 cells were cultured in Dulbecco’s modified Eagle’s medium (DMEM; 4.5 g/liter of glucose, Invitrogen, 4165-062)/Medium 199 (Invitrogen, 22340-020) (4:1), supplemented with 20% fetal bovine serum (FBS), fetuin (25 μg/ml), human insulin (5 μg/ml), gentamycin (40 μg/ml), and basic fibroblast growth factor (0.5 ng/ml) along with human epidermal growth factor (5 ng/ml) and dexamethasone (0.2 μg/ml) (*63*). Cells were maintained at 37°C in a humidified atmosphere with 5% CO_2_. Cells were split at 70 to 80% confluence, and the medium was replaced every48 hours.

To induce differentiation, LHCN-M2 and C79 cells were seeded at 90% confluence and switched at confluence to differentiation medium [DMEM supplemented with 2% horse serum and gentamycin (40 μg/ml)] for up to 5 days, with medium changes every 48 hours. For transfection, human AR small interfering RNA (siRNA; 5′-GGGCACTTCGACCATTTCTGA-3′), or scramble siRNA (5′-AAGCTTCATAAGGCGCATAGC-3′), were introduced using Typical RNAiMAX Transfection kit (Thermo Fisher Scientific, 13778075), following the manufacturer’s protocol. Cells were seeded in six-well plate and transfected with 50 nM siRNA diluted in Opti-MEM (Gibco, USA) at 50 to 60% confluence for 24 hours, after which the medium was replaced with fresh differentiation or growth medium. Cells were subsequently treated with 100 nM of DHT (Sigma-Aldrich, A8380-1G) diluted in ethanol 24 hours after transfections.

For AR overexpression, MuSC were transfected with the pEGFP-AR^WT^ plasmid using Lipofectamine 2000 DNA Transfection kit (Thermo Fisher Scientific, 11668027), according to the manufacturer’s instructions. Cells were seeded in six-well plate and transfected with 14 μg of pEGFP-AR^WT^ plasmid diluted in Opti-MEM (Gibco, USA) at 50 to 60% confluence for 24 hours, after which the medium was replaced with fresh differentiation or growth medium. Cells were subsequently treated with 100 nM of DHT (Sigma-Aldrich, A8380-1G) diluted in ethanol 24 hours after transfections.

#### Cell proliferation analysis

Cell proliferation was assessed using the CellTrace CFSE (carboxyfluorescein succinimidyl ester) Assay (Thermo Fisher Scientific, USA, C34557) as described (*64*). Briefly, LHCN-M2 and C79 cells were incubated with 5 μM CFSE at 37°C for 20 min in a humidified atmosphere with 5% CO_2_. The labeling reaction was quenched by adding an equal volume of complete growth medium, followed by two washes with PBS to remove excess dye. Labeled cells were kept in appropriate culture conditions and collected at 24, 48, and 72 hours postlabeling for flow cytometry analysis. CFSE fluorescence intensity was measured using a flow cytometer (BD LSRFortessa, BD Biosciences, USA) with excitation at 488 nm and emission detection at 530 nm. Data were analyzed using the FlowJo software (BD Biosciences, USA), and cell proliferation was determined by monitoring the progressive dilution of CFSE fluorescence across multiple cell generations. To identify non- and slow-proliferating cells, we defined the 30% of cells exhibiting the highest CellTrace Violet signal in control as the reference population and calculated the proportion of cells with equal or greater signal intensity.

#### Immunocytochemistry

Immunocytofluorescence assay was performed by incubating fixed LHCN-M2 and C79 cells with AR (rabbit, Abcam, ab108341; 1:500) and PAX7 (mouse, DSHB, AB_528428; 1:50) antibodies. Observations were made under a Leica confocal microscope.

#### Seahorse analysis of LHCN-M2 myoblasts

Mitochondrial respiration was assessed using the Seahorse XF Cell Mito Stress Test Kit (Agilent Technologies, part number: 103015-100) according to the manufacturer’s protocol. Briefly, ∼1800 LHCN-M2 myoblasts were seeded in Seahorse XF cell culture microplates (Agilent) and treated with either vehicle or 100 nM flutamide (Abcam, ab141234) for 1 hour. The assay was performed on a Seahorse XFe96 Analyzer (Agilent), following the sequential injection of oligomycin (1 μM), carbonyl cyanide *p*-trifluoromethoxyphenylhydrazone (FCCP; 2.5 μM), and rotenone/antimycin A (1 μM). Basal respiration, adenosine 5′-triphosphate (ATP)–linked respiration, maximal respiration, and spare respiratory capacity were calculated using the Wave software (Agilent).

### Flow cytometry analysis

#### Muscle cell population composition

Muscle stromal vascular fraction was isolated as described (*65*). Briefly, hind limb muscles were dissected, minced into small fragments, and enzymatically digested with Dispase II (1.25 U/ml; STEMCELL Technologies, Cat: 07913) and Collagenase Type I (500 μg/ml; Thermo Fisher Scientific, 17018029). The resulting cell suspension was sequentially filtered through 100-, 70-, and 40-μm cell strainers (Corning Life Sciences) to remove debris. Cells isolated from digested muscle tissue were stained with CD45 Alexa eFluor 700 (eBioscience, 56-0451-82; 1:100), CD31 PE (eBioscience, 12-0311-82; 1:100), SCA-1 PE-Cy7 (eBioscience, 25-5981-82; 1:100), and Integrin-α_7_ (ITGΑ7) fluorescein isothiocyanate (FITC; MBL, K0046-4; 1:50) antibodies. Cells were analyzed on the basis of forward scatter (FSC) and side scatter (SSC) parameters to exclude debris and doublets. CD45- and CD31-positive cells were quantified to assess immune and endothelial cell populations, respectively. After removing CD45/CD31-expressing cells, SCA-1–positive cells were gated to identify FAP cells. Last, SCA-1–negative, ITGΑ7-positive cells were classified as satellite cells. Fluorescence intensity was measured using a flow cytometer (BD FACSymphony A1, BD Biosciences, USA). Analysis were conducted using the FlowJO software as described (*65*).

#### Muscle immune cell population composition

Cells isolated from digested muscle tissue were stained with CD45 Alexa eFluor 700 (eBioscience, 56-0451-82; 1:100), CD11b PerCP-Cy5.5 (BioLegend, 101228; 1:100), Ly-6G-GR-1 FITC 488 (BioLegend, 108417; 1:100), Ly-6C PE-CF594 (BD Horizon, AB_2737749; 1:100), and F4/80 APC eFluor 780 (eBioscience, 47-4801-82; 1:100) antibodies. Cells were analyzed on the basis of FSC and SSC parameters to exclude debris and doublets.

Data analysis was performed by sequential gating on single, live CD45^+^ leukocytes, followed by identification of myeloid cells as CD11b^+^, neutrophils as CD11b^+^Ly6G^+^, monocytes as CD11b^+^Ly6G^−^Ly6C^+^, and macrophages as CD11b^+^Ly6G^−^ Ly6C^−^F4/80^+^. Fluorescence intensity was measured using a flow cytometer (BD FACSymphony A1, BD Biosciences, USA). Analyses were conducted using the FlowJO software.

### FACS of satellite cells

After stromal vascular fraction isolation, mononucleated cells were pelleted by centrifugation at 400*g* for 5 min, resuspended in FACS buffer [DMEM without phenol red, glucose (4.5 g/liter), 2% bovine serum albumin, and 2 mM EDTA], and labeled with fluorescence-conjugated antibodies. Cells were stained with CD45 PE (eBioscience, 12-0451-83; 1:100), CD11b PE-Cy7 (eBioscience, 25-0112-82; 1:100), CD31 PE (eBioscience, 12-0311-82; 1:100), TER119 PE (BD Pharmingen, 553673; 1:100), ITGΑ7 FITC (MBL, K0046-4; 1:50), CXCR4 APC (eBioscience, 17-9991-82; 1:100), and CD34 eFluor 405 (eBioscience, 48-0341-82; 1:33) antibodies. Cells were gated on the basis of FSC and SSC parameters to exclude cellular debris and doublets. Leukocytes and endothelial cells were removed by gating out CD45-, CD11b-, CD31-, and TER119-positive populations. The remaining fraction was further gated for CD34 and ITGΑ7 expression, and double-positive cells were selected on the basis of CXCR4 expression. Fluorescence intensity and sorting were conducted using a FACS sorter (BD FACSAria, BD Biosciences, USA). Sorted satellite cells were subsequently processed for RNA extraction or CUT&RUN analyses.

### CUT&RUN assay and bioinformatics analysis

CUT&RUN was performed as described (*65*, *66*). Briefly, FACS-isolated satellite cells were centrifuged at 500*g* for 10 min at 4°C, and the resulting cell pellet was incubated with nuclear extraction buffer [20 mM Hepes-KOH (pH 7.9), 10 mM KCl, 0.5 mM spermidine, 0.1% Triton X-100, 20% glycerol, and protease inhibitor cocktail] for 20 min on ice. Isolated nuclei were then washed and incubated with Concanavalin A–coated beads (BioMag Plus Concanavalin A, catalog no. 86057-3) for 10 min at 4°C. Following additional washes, the nuclei-bead complexes were incubated overnight at 4°C with gentle agitation in the presence of primary antibodies targeting AR (Abcam, ab108341; 1:100), H3K4me2 (Active Motif, 39141; 1:100), and a rabbit IgG (PeproTech, 500-P000; 1:100). Nuclei-bead complexes were precipitated and incubated with protein A–micrococcal nuclease (IGBMC) for 1 hour at 4°C under constant agitation. Enzymatic cleavage was initiated by adding 3 μl of 100 mM CaCl_2_, followed by 30 min of incubation on ice. The reaction was terminated at 37°C for 20 min using stop buffer [200 mM NaCl, 20 mM EDTA, 4 mM EGTA, ribonuclease A (50 μg/ml), glycogen (40 μg/ml), yeast spike-in DNA (25 pg/ml)]. DNA was extracted from immunoprecipitated samples for sequencing.

Libraries were generated from immunoprecipitated DNA as described (*65*) and sequenced on an Illumina NextSeq 2000 platform. Reads overlapping ENCODE blacklist regions (V2) were removed, and deduplicated unique reads were conserved for further analysis. Reads were mapped to the mm10 reference genome using Bowtie2 (v2.3.4.3) (*67*). To generate genome-wide intensity profiles, uniquely mapped reads were processed as follows: Bigwig files were generated using bamCoverage (deepTools 3.3.0) (*68*), with normalization by RPKM (--normalizeUsing RPKM --binSize 20). Raw bedgraph files were produced with genomeCoverageBed (BEDTools v2.26.0) (*69*). Peak calling was performed using SEACR (*70*) and MACS2 (*71*) with IgG-immunoprecipitated samples as controls (-keepall), and peaks with an false discovery rate (FDR) at least less than 0.05 were retained for further analysis. The IGV genome browser (http://software.broadinstitute.org/software/igv/) was used for genome-wide intensity visualization. Peaks were annotated, and motif analysis was conducted using HOMER. Genomic feature distribution (promoters/TSS, 5′ untranslated regions (5′UTRs), exons, introns, 3′UTRs, transcription termination sites, and intergenic regions) was determined on the basis of RefSeq and HOMER annotations. For bioinformatics and clustering analysis, Venn diagrams were generated using Venny (2.1.0) (https://bioinfogp.cnb.csic.es/tools/venny/).

Clustering analyses were performed using seqMINER (*72*) and deepTools (-computeMatrix -skip0; -plotHeatmap) with K-Means linear normalization. Genomic intersections and sequence extractions were carried out using BEDTools. GetFastaBed was used to extract FASTA sequences from BED files. Intersect Interval and Multiple Intersect were applied to identify overlapping genomic locations. WindowBed was used for gene-centric peak distribution analyses. When not specified, all bioinformatics analyses were performed using default parameters, ensuring robust and reproducible results.

All sequencing datasets generated in this study have been deposited in the Gene Expression Omnibus (GEO) under the following accession numbers: GSE303615 for CUT&RUN analysis at 5 dpi, GSE303618 for CUT&RUN analysis at 7 dpi, and GSE303620 for CUT&RUN analysis in NI animals.

### RNA extraction and analyses

Total RNA from muscle tissue, LHCN-M2, and C79 cell lines was extracted using TRIzol reagent (TRI Reagent, Molecular Research Center, TR-118, 513-841-0900) as described (*73*). RNA concentration and purity were assessed spectrophotometrically using a NanoDrop system (Thermo Fisher Scientific). For RNA isolation from FACS-isolated MuSC, the RNeasy Plus Micro Kit (QIAGEN, 74034) was used according to the manufacturer’s instructions.

For reverse transcription quantitative PCR (RT-qPCR) experiments, 2 μg of total RNA underwent reverse transcription using SuperScript IV (Life Technologies) with oligo(dT) primers, according to the supplier’s protocol. cDNA was diluted hundred times, and qPCR was performed with a LightCycler 480 II (Roche) using the SYBR Green PCR kit (Roche), in accordance with the recommended protocol (2 μl of cDNA, 4.8 μl of H_2_O, 5 μl of Syber Green 2× mix, and 0.2 μl of 100 μM primer mix). The primer sequences for mouse and human are detailed in tables S5 and S6, respectively. Housekeeping genes *Tbp* (for mouse) and *HSP90AB1* (for human) were used as internal controls. Data were analyzed using ∆∆*C*_t_ (*74*) method.

For RNA-seq and SMART-seq experiments, RNA integrity was assessed using a Bioanalyzer. Library preparation was performed at the GenomEast platform from IGBMC.

For RNA-seq, cDNA library was prepared and sequenced by the standard Illumina protocol [HiSeq 4000, single-end, 50 base pairs (bp)], following the manufacturer’s instructions. Image analysis and base calling were performed using RTA 2.7.7 and bcl2fastq 2.17.1.14. Adapter dimer reads were removed using DimerRemover. FastQC 0.11.2 (www.bioinformatics.babraham.ac.uk/projects/fastqc/) was used to evaluate the quality of the sequencing. Reads were mapped to the mouse mm10 genome (National Center for Biotechnology Information Build 38) using STAR v2.7.10b (*75*). Only uniquely aligned reads were retained for further analyses. Quantification of gene expression was performed using HTSeq 0.11.0 (*76*). For comparison among datasets, the transcripts with more than 100 raw reads were considered.

For SMART-seq, full-length cDNA was generated from 250 pg to 2 ng of total RNA using SMART-Seq mRNA Kit (Takara Bio Europe, Saint Germain en Laye, France), according to the manufacturer’s instructions, with 12 cycles of PCR for cDNA amplification by SeqAmp polymerase. Six hundred picograms of preamplified cDNA was used as input for transposon 5 tagmentation, followed by 12 cycles of library amplification using Nextera XT DNA Library Preparation Kit and IDT for Illumina DNA/RNA UD Indexes, Tagmentation (Illumina, San Diego, USA). Following purification with SPRIselect beads (Beckman Coulter, Villepinte, France), the size and concentration of libraries were assessed using Bioanalyzer 2100 system (Agilent technologies, Les Ulis, France). Libraries were sequenced on an Illumina NextSeq 2000-X sequencer as paired-end 50 base reads. Image analysis and base calling were performed using RTA version 2.7.7 and BCL Convert version 3.8.4. Adapter dimer reads were removed with DimerRemover (-a AGATCGGAAGAGCACACGTCTGAACTCCAGTCAC), and the quality of sequencing data was evaluated using FastQC 0.11.5. Reads were aligned to the mouse mm39 genome using STAR v2.7.10b, retaining only uniquely mapped reads for downstream analyses. Gene expression was quantified using STAR, and for dataset comparisons, only transcripts with more than 50 raw reads were considered.

For RNA-seq and SMART-seq experiments, DEGs were identified using the Bioconductor libraries DESeq2 (*77*) with *P* < 0.05. Identified DEGs were subjected to functional analysis using STRING 12.0 (*78*) and to pathway analysis using WebGestalt (*79*) with the overrepresentation analysis method, applying an FDR < 0.05 as the significance threshold. Heatmaps were generated by centering and normalizing expression values using Cluster 3.0 (*80*), with subsequent visualization Morpheus (https://software.broadinstitute.org/morpheus/). Genes were clustered using K-Mean with gene tree construction, Pearson correlation, and average linkage as clustering parameters (*81*). All sequencing datasets generated in this study have been deposited in the GEO under the following accession numbers: GSE303612 for SMART-seq and GSE303614 for bulk RNA-seq.

### Single-cell RNA sequencing

scRNA-seq experiments were performed on TA muscle cells, isolated from three control (AR^L2/Y^) and three AR^(i)sat-/Y^/YFP^Tg/0^ injured TA muscles at 5 dpi. TA muscles were dissociated into single cells, as described above. Cells from each genotype group were pooled and stained with DAPI, and DAPI-negative living cells were isolated using a BD FACS Aria Fusion flow cytometer. To assess cell viability and yield, a Trypan blue exclusion assay was performed using a Neubauer chamber. Only samples with a viability greater than 95% were processed using a Chromium Controller (10x Genomics, Leiden, The Netherlands). Total cells (16,500) were loaded per well to yield approximately 10,000 captured cells into nanoliter-scale Gel Beads-in-Emulsion (GEMs). Single-cell 3′ mRNA sequencing library was generated using Chromium Next GEM Single Cell 3′ prime Reagent Kits v3.1 (10x Genomics). Libraries were quantified and controlled for their quality using Bioanalyzer 2100 (Agilent Technologies, Santa Clara CA) and further sequenced on an Illumina NextSeq 550 sequencer as paired-end 28 + 100 base reads. Image analysis and base calling were performed using RTA version 2.7.7 (*82*) and Cell Ranger version 3.0.2 (*83*).

Data were processed using the Cell Ranger 7.0.0 count pipeline (10x Genomics) for read alignment, barcode and unique molecular identifier (UMI) filtering, and gene quantification. A custom reference genome was generated using the mkref function with the GRCm39 assembly of the *Mus musculus* genome and Ensembl release 107 annotations. The output gene-barcode matrix was imported into RStudio (version 2025.05.1) using the Read10X function from the Seurat package (version 5), yielding a sparse matrix of UMI counts per gene per cell. Downstream analyses were conducted in RStudio following standard Seurat v5 workflows (*84*), including quality control, normalization, dimensionality reduction, clustering, and differential gene expression. For quality control, cells with <10% mitochondrial content, <50% ribosomal gene expression, and total RNA features between 200 and 6000 were retained. Principal components analysis (dim = 16) was used for dimensionality reduction, and clustering was performed using a resolution parameter of 0.6. Differential gene expression and further downstream analyses were conducted. Satellite cell reclustering was performed with dimension reduction of 14 and cluster resolution of 0.3.

Cell-cell communication analysis was conducted using the CellChat R package within the RStudio environment (*85*). Normalized scRNA-seq data were used, and overexpressed ligands and receptors were identified to infer intercellular signaling networks. Communication probabilities were computed using the trimean method, with a minimum gene expression threshold of 0.1, and genes were considered only if expressed in more than 10% of cells in the described groups. The CellChatDB ligand-receptor interaction database was used as the reference. The analysis pipeline included identification of major signaling inputs and outputs, classification of signaling roles (incoming, outgoing, mediator, and influencer), and visualization via circle plots and hierarchical clustering. Intercondition comparisons were performed using the compareInteractions and rankNet functions. All analyses were performed using default CellChat parameters, including 500 permutations for significance testing and standard visualization settings, unless otherwise specified. Results were visualized and further interpreted using built-in and custom R scripts. Cell cycle bioinformatic analyses were performed using Scanpy function (scanpy.tl.score_genes_cell_cycle), with marker genes extracted from scRNA-seq of mouse embryonic stem cells generated for DeepCycle as described (*84*, *86*). All sequencing datasets generated in this study have been deposited in the GEO under the following accession numbers: GSE303611 for scRNA-seq.

### Protein analysis

TA muscles were mechanically ground in radioimmunoprecipitation assay (RIPA) buffer [50 mM tris (pH 7.5), 1% NP-40, 0.5% sodium deoxycholate, 0.1% SDS, 150 mM NaCl, 5 mM EDTA, and a protease inhibitor cocktail (45 mg/ml; Roche, catalog no. 11 873 580 001)] at 4°C. Alternatively, adherent cells were harvested in RIPA buffer and kept on ice for 10 min. TA muscles and cell lysates were centrifuged at 12,000*g* for 10 min at 4°C, and the supernatants were collected for further analysis. For Western blot analyses, homogenates were separated in 8% polyacrylamide gels, transferred to Hybond nitrocellulose membranes (Amersham Biosciences), and probed with specific antibodies targeting AR (Abcam, ab108341; 1:500), α-TUBULIN (IGBMC, 1Tub2A2; 1:5000), and glyceraldehyde-3-phosphate dehydrogenase (GAPDH; Cell Signaling Techology, #2118; 1:5000). Secondary antibodies conjugated to horseradish peroxidase [anti-Mouse-HPR, Cell Signaling Technology, 7076S (1:10,000); anti-Rabbit-HPR, Cell Signaling Technology, 7074S (1:10,000)] were detected using chemiluminescence detection systems (GE Healthcare, Amersham, ImageQuant 800 or Imager 600). Protein quantification was assessed by the FIJI/ImageJ distribution software (https://imagej.net/ImageJ) (*87*).

### Statistical analyses

Data processing and analysis were conducted using DESeq2, Microsoft Excel, and GraphPad Prism 9. In all graphical representations, error bars indicate the SEM. Comparisons between groups were performed using appropriate statistical tests, selected on the basis of the number of groups, data distribution (normality), variance homogeneity, and multiple comparisons. The specific statistical tests applied are indicated in the figure legends. Statistical significance was defined as follows: n.s., *P* ≥ 0.05 (not significant); *P < 0.05; **P < 0.01; and ***P < 0.001.

## References

[1] S. B. Charge, M. A. Rudnicki, Cellular and molecular regulation of muscle regeneration. Physiol. Rev. 84, 209–238 (2004). 14715915 10.1152/physrev.00019.2003

[2] A. J. Goel, M. K. Rieder, H. H. Arnold, G. L. Radice, R. S. Krauss, Niche cadherins control the quiescence-to-activation transition in muscle stem cells. Cell Rep. 21, 2236–2250 (2017). 29166613 10.1016/j.celrep.2017.10.102 PMC5702939

[3] M. Rozo, L. Li, C. M. Fan, Targeting β1-integrin signaling enhances regeneration in aged and dystrophic muscle in mice. Nat. Med. 22, 889–896 (2016). 27376575 10.1038/nm.4116 PMC4974124

[4] P. Seale, L. A. Sabourin, A. Girgis-Gabardo, A. Mansouri, P. Gruss, M. A. Rudnicki, Pax7 is required for the specification of myogenic satellite cells. Cell 102, 777–786 (2000). 11030621 10.1016/s0092-8674(00)00066-0

[5] Y. X. Wang, M. A. Rudnicki, Satellite cells, the engines of muscle repair. Nat. Rev. Mol. Cell Biol. 13, 127–133 (2012). 10.1038/nrm3265 22186952

[6] J. E. Anderson, Key concepts in muscle regeneration: Muscle “cellular ecology” integrates a gestalt of cellular cross-talk, motility, and activity to remodel structure and restore function. Eur. J. Appl. Physiol. 122, 273–300 (2022). 34928395 10.1007/s00421-021-04865-4 PMC8685813

[7] W. Zhang, Y. Liu, H. Zhang, Extracellular matrix: An important regulator of cell functions and skeletal muscle development. Cell Biosci. 11, 65 (2021). 33789727 10.1186/s13578-021-00579-4 PMC8011170

[8] H. Koike, I. Manabe, Y. Oishi, Mechanisms of cooperative cell-cell interactions in skeletal muscle regeneration. Inflamm. Regen. 42, 48 (2022). 36380396 10.1186/s41232-022-00234-6 PMC9667595

[9] R. Abou-Khalil, F. le Grand, G. Pallafacchina, S. Valable, F. J. Authier, M. A. Rudnicki, R. K. Gherardi, S. Germain, F. Chretien, A. Sotiropoulos, P. Lafuste, D. Montarras, B. Chazaud, Autocrine and paracrine angiopoietin 1/Tie-2 signaling promotes muscle satellite cell self-renewal. Cell Stem Cell 5, 298–309 (2009). 19733541 10.1016/j.stem.2009.06.001 PMC4592285

[10] V. R. Kedlian, Y. Wang, T. Liu, X. Chen, L. Bolt, C. Tudor, Z. Shen, E. S. Fasouli, E. Prigmore, V. Kleshchevnikov, J. P. Pett, T. Li, J. E. G. Lawrence, S. Perera, M. Prete, N. Huang, Q. Guo, X. Zeng, L. Yang, K. Polański, N. J. Chipampe, M. Dabrowska, X. Li, O. A. Bayraktar, M. Patel, N. Kumasaka, K. T. Mahbubani, A. P. Xiang, K. B. Meyer, K. Saeb-Parsy, S. A. Teichmann, H. Zhang, Human skeletal muscle aging atlas. Nat. Aging 4, 727–744 (2024). 38622407 10.1038/s43587-024-00613-3 PMC11108788

[11] J. Su, C. Ekman, N. Oskolkov, L. Lahti, K. Ström, A. Brazma, L. Groop, J. Rung, O. Hansson, A novel atlas of gene expression in human skeletal muscle reveals molecular changes associated with aging. Skelet. Muscle 5, 35 (2015). 26457177 10.1186/s13395-015-0059-1 PMC4600214

[12] V. Moiseeva, A. Cisneros, V. Sica, O. Deryagin, Y. Lai, S. Jung, E. Andrés, J. An, J. Segalés, L. Ortet, V. Lukesova, G. Volpe, A. Benguria, A. Dopazo, S. A. Benitah, Y. Urano, A. del Sol, M. A. Esteban, Y. Ohkawa, A. L. Serrano, E. Perdiguero, P. Muñoz-Cánoves, Senescence atlas reveals an aged-like inflamed niche that blunts muscle regeneration. Nature 613, 169–178 (2023). 36544018 10.1038/s41586-022-05535-x PMC9812788

[13] K. Day, G. Shefer, A. Shearer, Z. Yablonka-Reuveni, The depletion of skeletal muscle satellite cells with age is concomitant with reduced capacity of single progenitors to produce reserve progeny. Dev. Biol. 340, 330–343 (2010). 20079729 10.1016/j.ydbio.2010.01.006 PMC2854302

[14] F. Petermann-Rocha, V. Balntzi, S. R. Gray, J. Lara, F. K. Ho, J. P. Pell, C. Celis-Morales, Global prevalence of sarcopenia and severe sarcopenia: A systematic review and meta-analysis. J. Cachexia. Sarcopenia Muscle 13, 86–99 (2022). 34816624 10.1002/jcsm.12783 PMC8818604

[15] P. Munoz-Canoves, J. Neves, P. Sousa-Victor, Understanding muscle regenerative decline with aging: New approaches to bring back youthfulness to aged stem cells. FEBS J. 287, 406–416 (2020). 31854082 10.1111/febs.15182

[16] J. Rizk, R. Sahu, D. Duteil, An overview on androgen-mediated actions in skeletal muscle and adipose tissue. Steroids 199, 109306 (2023). 37634653 10.1016/j.steroids.2023.109306

[17] I. M. Conboy, T. A. Rando, The regulation of Notch signaling controls satellite cell activation and cell fate determination in postnatal myogenesis. Dev. Cell 3, 397–409 (2002). 12361602 10.1016/s1534-5807(02)00254-x

[18] J. Y. Seo, J. H. Kim, Y. Y. Kong, Unraveling the paradoxical action of androgens on muscle stem cells. Mol. Cells 42, 97–103 (2019). 30759971 10.14348/molcells.2019.0004 PMC6399011

[19] L. Niel, K. R. Willemsen, S. N. Volante, D. A. Monks, Sexual dimorphism and androgen regulation of satellite cell population in differentiating rat levator ani muscle. Dev. Neurobiol. 68, 115–122 (2008). 17948238 10.1002/dneu.20580

[20] P. Diel, D. Baadners, K. Schlupmann, M. Velders, J. P. Schwarz, C2C12 myoblastoma cell differentiation and proliferation is stimulated by androgens and associated with a modulation of myostatin and Pax7 expression. J. Mol. Endocrinol. 40, 231–241 (2008). 18434429 10.1677/JME-07-0175

[21] Y. Chen, J. D. Zajac, H. E. MacLean, Androgen regulation of satellite cell function. J. Endocrinol. 186, 21–31 (2005). 16002532 10.1677/joe.1.05976

[22] A. Klose, W. Liu, N. D. Paris, S. Forman, J. J. Krolewski, K. L. Nastiuk, J. V. Chakkalakal, Castration induces satellite cell activation that contributes to skeletal muscle maintenance. JCSM Rapid Commun. 1, 1–16 (2018). PMC5959044 29782610

[23] H. Sakai, Y. Imai, Cell-specific functions of androgen receptor in skeletal muscles. Endocr. J. 71, 437–445 (2024). 38281756 10.1507/endocrj.EJ23-0691

[24] I. Sinha-Hikim, W. E. Taylor, N. F. Gonzalez-Cadavid, W. Zheng, S. Bhasin, Androgen receptor in human skeletal muscle and cultured muscle satellite cells: Up-regulation by androgen treatment. J. Clin. Endocrinol. Metab. 89, 5245–5255 (2004). 15472231 10.1210/jc.2004-0084

[25] K. Ghaibour, M. Schuh, S. Souali-Crespo, C. Chambon, A. Charlot, J. Rizk, D. Rovito, A. I. Rerra, Q. Cai, N. Messaddeq, J. Zoll, D. Duteil, D. Metzger, Androgen receptor coordinates muscle metabolic and contractile functions. J. Cachexia. Sarcopenia Muscle 14, 1707–1720 (2023). 37208984 10.1002/jcsm.13251 PMC10401547

[26] C. Chambon, D. Duteil, A. Vignaud, A. Ferry, N. Messaddeq, R. Malivindi, S. Kato, P. Chambon, D. Metzger, Myocytic androgen receptor controls the strength but not the mass of limb muscles. Proc. Natl. Acad. Sci. U.S.A. 107, 14327–14332 (2010). 20660752 10.1073/pnas.1009536107 PMC2922552

[27] P. Mourikis, S. Tajbakhsh, Distinct contextual roles for Notch signalling in skeletal muscle stem cells. BMC Dev. Biol. 14, 2 (2014). 24472470 10.1186/1471-213X-14-2 PMC3903015

[28] F. X. Pizza, K. H. Buckley, Regenerating myofibers after an acute muscle injury: What do we really know about them? Int. J. Mol. Sci. 24, 12545 (2023). 37628725 10.3390/ijms241612545 PMC10454182

[29] S. N. Oprescu, F. Yue, J. Qiu, L. F. Brito, S. Kuang, Temporal dynamics and heterogeneity of cell populations during skeletal muscle regeneration. iScience 23, 100993 (2020). 32248062 10.1016/j.isci.2020.100993 PMC7125354

[30] D. W. McKellar, L. D. Walter, L. T. Song, M. Mantri, M. F. Z. Wang, I. de Vlaminck, B. D. Cosgrove, Large-scale integration of single-cell transcriptomic data captures transitional progenitor states in mouse skeletal muscle regeneration. Commun. Biol. 4, 1280 (2021). 34773081 10.1038/s42003-021-02810-x PMC8589952

[31] F. Yue, S. N. Oprescu, J. Qiu, L. Gu, L. Zhang, J. Chen, N. Narayanan, M. Deng, S. Kuang, Lipid droplet dynamics regulate adult muscle stem cell fate. Cell Rep. 38, 110267 (2022). 35045287 10.1016/j.celrep.2021.110267 PMC9127130

[32] J. Morroni, A. Benedetti, L. Esposito, M. de Bardi, G. Borsellino, C. S. Riera, L. Giordani, M. Bouche, B. Lozanoska-Ochser, Injury-experienced satellite cells retain long-term enhanced regenerative capacity. Stem Cell Res. Ther. 14, 246 (2023). 37697344 10.1186/s13287-023-03492-4 PMC10496398

[33] C. E. Brun, M. C. Sincennes, A. Y. T. Lin, D. Hall, W. Jarassier, P. Feige, F. le Grand, M. A. Rudnicki, GLI3 regulates muscle stem cell entry into G_Alert_ and self-renewal. Nat. Commun. 13, 3961 (2022). 35803939 10.1038/s41467-022-31695-5 PMC9270324

[34] S. T. Barsky, D. A. Monks, The role of androgens and global and tissue-specific androgen receptor expression on body composition, exercise adaptation, and performance. Biol. Sex Differ. 16, 28 (2025). 40269952 10.1186/s13293-025-00707-6 PMC12016402

[35] G. Chinvattanachot, D. Rivas, G. Duque, Mechanisms of muscle cells alterations and regeneration decline during aging. Ageing Res. Rev. 102, 102589 (2024). 39566742 10.1016/j.arr.2024.102589

[36] C. Serra, F. Tangherlini, S. Rudy, D. Lee, G. Toraldo, N. L. Sandor, A. Zhang, R. Jasuja, S. Bhasin, Testosterone improves the regeneration of old and young mouse skeletal muscle. J. Gerontol. A Biol. Sci. Med. Sci. 68, 17–26 (2013). 22499765 10.1093/gerona/gls083 PMC3598367

[37] M. Oura, B. K. Son, Z. Song, K. Toyoshima, M. Nanao-Hamai, S. Ogawa, M. Akishita, Testosterone/androgen receptor antagonizes immobility-induced muscle atrophy through Inhibition of myostatin transcription and inflammation in mice. Sci. Rep. 15, 10568 (2025). 40148525 10.1038/s41598-025-95115-6 PMC11950163

[38] Y. Lai, I. Ramírez-Pardo, J. Isern, J. An, E. Perdiguero, A. L. Serrano, J. Li, E. García-Domínguez, J. Segalés, P. Guo, V. Lukesova, E. Andrés, J. Zuo, Y. Yuan, C. Liu, J. Viña, J. Doménech-Fernández, M. C. Gómez-Cabrera, Y. Song, L. Liu, X. Xu, P. Muñoz-Cánoves, M. A. Esteban, Multimodal cell atlas of the ageing human skeletal muscle. Nature 629, 154–164 (2024). 38649488 10.1038/s41586-024-07348-6 PMC11062927

[39] V. Dubois, M. Laurent, S. Boonen, D. Vanderschueren, F. Claessens, Androgens and skeletal muscle: Cellular and molecular action mechanisms underlying the anabolic actions. Cell. Mol. Life Sci. 69, 1651–1667 (2012). 22101547 10.1007/s00018-011-0883-3 PMC11115174

[40] R. L. Sheppard, E. E. Spangenburg, E. R. Chin, S. M. Roth, Androgen receptor polyglutamine repeat length affects receptor activity and C2C12 cell development. Physiol. Genomics 43, 1135–1143 (2011). 21828246 10.1152/physiolgenomics.00049.2011 PMC3217329

[41] S. Fu, X. Lin, L. Yin, X. Wang, Androgen receptor regulates the proliferation of myoblasts under appropriate or excessive stretch through IGF-1 receptor mediated p38 and ERK1/2 pathways. Nutr. Metab. 18, 85 (2021). 10.1186/s12986-021-00610-y PMC8444398 34526063

[42] S. Fu, J. Hu, G. Wang, Z. Qian, X. Wang, Androgen receptor regulates the differentiation of myoblasts under cyclic mechanical stretch and its upstream and downstream signals. Int. J. Biol. Macromol. 281, 136257 (2024). 39366623 10.1016/j.ijbiomac.2024.136257

[43] V. Dubois, M. R. Laurent, F. Jardi, L. Antonio, K. Lemaire, L. Goyvaerts, L. Deldicque, G. Carmeliet, B. Decallonne, D. Vanderschueren, F. Claessens, Androgen deficiency exacerbates high-fat diet-induced metabolic alterations in male mice. Endocrinology 157, 648–665 (2016). 26562264 10.1210/en.2015-1713

[44] A. Rocchi, C. Milioto, S. Parodi, A. Armirotti, D. Borgia, M. Pellegrini, A. Urciuolo, S. Molon, V. Morbidoni, M. Marabita, V. Romanello, P. Gatto, B. Blaauw, P. Bonaldo, F. Sambataro, D. M. Robins, A. P. Lieberman, G. Sorarù, L. Vergani, M. Sandri, M. Pennuto, Glycolytic-to-oxidative fiber-type switch and mTOR signaling activation are early-onset features of SBMA muscle modified by high-fat diet. Acta Neuropathol. 132, 127–144 (2016). 26971100 10.1007/s00401-016-1550-4 PMC4911374

[45] M. D. Campbell, D. Djukovic, D. Raftery, D. J. Marcinek, Age-related changes of skeletal muscle metabolic response to contraction are also sex-dependent. J. Physiol. 603, 69–86 (2025). 37742081 10.1113/JP285124 PMC10959763

[46] L. Grevendonk, N. J. Connell, C. McCrum, C. E. Fealy, L. Bilet, Y. M. H. Bruls, J. Mevenkamp, V. B. Schrauwen-Hinderling, J. A. Jörgensen, E. Moonen-Kornips, G. Schaart, B. Havekes, J. de Vogel-van den Bosch, M. C. E. Bragt, K. Meijer, P. Schrauwen, J. Hoeks, Impact of aging and exercise on skeletal muscle mitochondrial capacity, energy metabolism, and physical function. Nat. Commun. 12, 4773 (2021). 34362885 10.1038/s41467-021-24956-2 PMC8346468

[47] A. Kobayashi, K. Azuma, K. Ikeda, S. Inoue, Mechanisms underlying the regulation of mitochondrial respiratory chain complexes by nuclear steroid receptors. Int. J. Mol. Sci. 21, 6683 (2020). 32932692 10.3390/ijms21186683 PMC7555717

[48] K. Jomova, R. Raptova, S. Y. Alomar, S. H. Alwasel, E. Nepovimova, K. Kuca, M. Valko, Reactive oxygen species, toxicity, oxidative stress, and antioxidants: Chronic diseases and aging. Arch. Toxicol. 97, 2499–2574 (2023). 37597078 10.1007/s00204-023-03562-9 PMC10475008

[49] J. Lee, Y. H. Lin, P. K. Chiang, J. B. Lin, Y. T. Jan, W. K. Tsai, Y. J. Chen, K. P. Wu, Androgen deprivation therapy-induced muscle loss and fat gain predict cardiovascular events in prostate cancer patients. J. Cachexia. Sarcopenia Muscle 16, e13844 (2025). 40464195 10.1002/jcsm.13844 PMC12134783

[50] J. M. Argiles, N. Campos, J. M. Lopez-Pedrosa, R. Rueda, L. Rodriguez-Manas, Skeletal muscle regulates metabolism via interorgan crosstalk: Roles in health and disease. J. Am. Med. Dir. Assoc. 17, 789–796 (2016). 27324808 10.1016/j.jamda.2016.04.019

[51] A. Penvose, J. L. Keenan, D. Bray, V. Ramlall, T. Siggers, Comprehensive study of nuclear receptor DNA binding provides a revised framework for understanding receptor specificity. Nat. Commun. 10, 2514 (2019). 31175293 10.1038/s41467-019-10264-3 PMC6555819

[52] L. Gelman, J. N. Feige, C. Tudor, Y. Engelborghs, W. Wahli, B. Desvergne, Integrating nuclear receptor mobility in models of gene regulation. Nucl. Recept. Signal. 4, e010 (2006). 16741568 10.1621/nrs.04010 PMC1472671

[53] G. L. Hager, A. K. Nagaich, T. A. Johnson, D. A. Walker, S. John, Dynamics of nuclear receptor movement and transcription. Biochim. Biophys. Acta 1677, 46–51 (2004). 15020044 10.1016/j.bbaexp.2003.09.016

[54] D. A. Stavreva, L. Hoang, K. Wagh, H. LaPoint, S. Kim, A. Upadhyaya, L. Schiltz, R. Chari, G. L. Hager, Nuclear receptors dynamics and gene regulation in response to ultradian hormone pulses. Endocrinology 166, bqaf043.034 (2025).

[55] M. Forouhan, W. F. Lim, L. C. Zanetti-Domingues, C. J. Tynan, T. C. Roberts, B. Malik, R. Manzano, A. A. Speciale, R. Ellerington, A. Garcia-Guerra, P. Fratta, G. Sorarú, L. Greensmith, M. Pennuto, M. J. A. Wood, C. Rinaldi, AR cooperates with SMAD4 to maintain skeletal muscle homeostasis. Acta Neuropathol. 143, 713–731 (2022). 35522298 10.1007/s00401-022-02428-1 PMC9107400

[56] S. R. Nath, Z. Yu, T. A. Gipson, G. B. Marsh, E. Yoshidome, D. M. Robins, S. V. Todi, D. E. Housman, A. P. Lieberman, Androgen receptor polyglutamine expansion drives age-dependent quality control defects and muscle dysfunction. J. Clin. Invest. 128, 3630–3641 (2018). 29809168 10.1172/JCI99042 PMC6063498

[57] F. J. Arnold, A. Pluciennik, D. E. Merry, Impaired nuclear export of polyglutamine-expanded androgen receptor in spinal and bulbar muscular atrophy. Sci. Rep. 9, 119 (2019). 30644418 10.1038/s41598-018-36784-4 PMC6333819

[58] H. Shiina, T. Matsumoto, T. Sato, K. Igarashi, J. Miyamoto, S. Takemasa, M. Sakari, I. Takada, T. Nakamura, D. Metzger, P. Chambon, J. Kanno, H. Yoshikawa, S. Kato, Premature ovarian failure in androgen receptor-deficient mice. Proc. Natl. Acad. Sci. U.S.A. 103, 224–229 (2006). 16373508 10.1073/pnas.0506736102 PMC1324980

[59] M. M. Murphy, J. A. Lawson, S. J. Mathew, D. A. Hutcheson, G. Kardon, Satellite cells, connective tissue fibroblasts and their interactions are crucial for muscle regeneration. Development 138, 3625–3637 (2011). 21828091 10.1242/dev.064162 PMC3152921

[60] M. Tosic, A. Allen, D. Willmann, C. Lepper, J. Kim, D. Duteil, R. Schüle, Lsd1 regulates skeletal muscle regeneration and directs the fate of satellite cells. Nat. Commun. 9, 366 (2018). 29371665 10.1038/s41467-017-02740-5 PMC5785540

[61] C. Chenu, M. Adlanmerini, F. Boudou, E. Chantalat, A. L. Guihot, C. Toutain, I. Raymond-Letron, P. Vicendo, A. P. Gadeau, D. Henrion, J. F. Arnal, F. Lenfant, Testosterone prevents cutaneous ischemia and necrosis in males through complementary estrogenic and androgenic actions. Arterioscler. Thromb. Vasc. Biol. 37, 909–919 (2017). 28360090 10.1161/ATVBAHA.117.309219

[62] D. Rovito, A. I. Rerra, V. Ueberschlag-Pitiot, S. Joshi, N. Karasu, V. Dacleu-Siewe, K. B. Rayana, K. Ghaibour, M. Parisotto, A. Ferry, S. A. Jelinsky, G. Laverny, B. P. Klaholz, T. Sexton, I. M. L. Billas, D. Duteil, D. Metzger, Myod1 and GR coordinate myofiber-specific transcriptional enhancers. Nucleic Acids Res. 49, 4472–4492 (2021). 33836079 10.1093/nar/gkab226 PMC8096230

[63] A. Simon, S. Djeddi, P. Bournon, D. Reiss, J. Thompson, J. Laporte, Transcriptomic characterization of postnatal muscle maturation. Dis. Model. Mech. 18, DMM052098 (2025). 39945189 10.1242/dmm.052098 PMC11911633

[64] M. Cigrang, J. Obid, M. Nogaret, L. Seno, T. Ye, G. Davidson, P. Catez, P. Berico, C. Capelli, C. Marechal, A. Zachayus, C. Elly, M. J. Guillen Navarro, M. Martinez Diez, G. Santamaria Nunez, T. K. Li, E. Compe, P. Avilés, I. Davidson, J. M. Egly, C. Cuevas, F. Coin, Pan-inhibition of super-enhancer-driven oncogenic transcription by next-generation synthetic ecteinascidins yields potent anti-cancer activity. Nat. Commun. 16, 512 (2025). 39779693 10.1038/s41467-024-55667-z PMC11711318

[65] K. Ghaibour, J. Rizk, C. Ebel, T. Ye, M. Philipps, V. Schreiber, D. Metzger, D. Duteil, An efficient protocol for CUT&RUN analysis of FACS-isolated mouse satellite cells. J. Vis. Exp., 10.3791/65215 (2023). 10.3791/65215 37486110

[66] D. H. Janssens, S. J. Wu, J. F. Sarthy, M. P. Meers, C. H. Myers, J. M. Olson, K. Ahmad, S. Henikoff, Automated in situ chromatin profiling efficiently resolves cell types and gene regulatory programs. Epigenetics Chromatin 11, 74 (2018). 30577869 10.1186/s13072-018-0243-8 PMC6302505

[67] B. Langmead, S. L. Salzberg, Fast gapped-read alignment with Bowtie 2. Nat. Methods 9, 357–359 (2012). 22388286 10.1038/nmeth.1923 PMC3322381

[68] F. Ramirez, F. Dundar, S. Diehl, B. A. Gruning, T. Manke, deepTools: A flexible platform for exploring deep-sequencing data. Nucleic Acids Res. 42, W187–W191 (2014). 24799436 10.1093/nar/gku365 PMC4086134

[69] A. R. Quinlan, I. M. Hall, BEDTools: A flexible suite of utilities for comparing genomic features. Bioinformatics 26, 841–842 (2010). 20110278 10.1093/bioinformatics/btq033 PMC2832824

[70] M. P. Meers, D. Tenenbaum, S. Henikoff, Peak calling by Sparse Enrichment Analysis for CUT&RUN chromatin profiling. Epigenetics Chromatin 12, 42 (2019). 31300027 10.1186/s13072-019-0287-4 PMC6624997

[71] Y. Zhang, T. Liu, C. A. Meyer, J. Eeckhoute, D. S. Johnson, B. E. Bernstein, C. Nusbaum, R. M. Myers, M. Brown, W. Li, X. S. Liu, Model-based analysis of ChIP-Seq (MACS). Genome Biol. 9, R137 (2008). 18798982 10.1186/gb-2008-9-9-r137 PMC2592715

[72] T. Ye, A. R. Krebs, M. A. Choukrallah, C. Keime, F. Plewniak, I. Davidson, L. Tora, seqMINER: An integrated ChIP-seq data interpretation platform. Nucleic Acids Res. 39, e35 (2011). 21177645 10.1093/nar/gkq1287 PMC3064796

[73] Q. Cai, R. Sahu, V. Ueberschlag-Pitiot, S. Souali-Crespo, C. Charvet, I. Silem, F. Cottard, T. Ye, F. Taleb, E. Metzger, R. Schuele, I. M. L. Billas, G. Laverny, D. Metzger, D. Duteil, LSD1 inhibition circumvents glucocorticoid-induced muscle wasting of male mice. Nat. Commun. 15, 3563 (2024). 38670969 10.1038/s41467-024-47846-9 PMC11053113

[74] K. J. Livak, T. D. Schmittgen, Analysis of relative gene expression data using real-time quantitative PCR and the 2^–ΔΔ*C*T^ method. Methods 25, 402–408 (2001). 11846609 10.1006/meth.2001.1262

[75] A. Dobin, C. A. Davis, F. Schlesinger, J. Drenkow, C. Zaleski, S. Jha, P. Batut, M. Chaisson, T. R. Gingeras, STAR: Ultrafast universal RNA-seq aligner. Bioinformatics 29, 15–21 (2013). 23104886 10.1093/bioinformatics/bts635 PMC3530905

[76] S. Anders, P. T. Pyl, W. Huber, HTSeq—A Python framework to work with high-throughput sequencing data. Bioinformatics 31, 166–169 (2015). 25260700 10.1093/bioinformatics/btu638 PMC4287950

[77] M. I. Love, W. Huber, S. Anders, Moderated estimation of fold change and dispersion for RNA-seq data with DESeq2. Genome Biol. 15, 550 (2014). 25516281 10.1186/s13059-014-0550-8 PMC4302049

[78] D. Szklarczyk, R. Kirsch, M. Koutrouli, K. Nastou, F. Mehryary, R. Hachilif, A. L. Gable, T. Fang, N. T. Doncheva, S. Pyysalo, P. Bork, L. J. Jensen, C. von Mering, The STRING database in 2023: Protein-protein association networks and functional enrichment analyses for any sequenced genome of interest. Nucleic Acids Res. 51, D638–D646 (2023). 36370105 10.1093/nar/gkac1000 PMC9825434

[79] J. Wang, D. Duncan, Z. Shi, B. Zhang, WEB-based GEne SeT AnaLysis Toolkit (WebGestalt): Update 2013. Nucleic Acids Res. 41, W77–W83 (2013). 23703215 10.1093/nar/gkt439 PMC3692109

[80] M. J. de Hoon, S. Imoto, J. Nolan, S. Miyano, Open source clustering software. Bioinformatics 20, 1453–1454 (2004). 14871861 10.1093/bioinformatics/bth078

[81] Y. Lu, S. Lu, F. Fotouhi, Y. Deng, S. J. Brown, Incremental genetic K-means algorithm and its application in gene expression data analysis. BMC Bioinformatics 5, 172 (2004). 15511294 10.1186/1471-2105-5-172 PMC543472

[82] A. Raine, U. Liljedahl, J. Nordlund, Data quality of whole genome bisulfite sequencing on Illumina platforms. PLOS ONE 13, e0195972 (2018). 29668744 10.1371/journal.pone.0195972 PMC5905984

[83] A. T. L. Lun, S. Riesenfeld, T. Andrews, T. P. Dao, T. Gomes, participants in the 1st Human Cell Atlas Jamboree, J. C. Marioni, EmptyDrops: Distinguishing cells from empty droplets in droplet-based single-cell RNA sequencing data. Genome Biol. 20, 63 (2019). 30902100 10.1186/s13059-019-1662-y PMC6431044

[84] R. Satija, J. A. Farrell, D. Gennert, A. F. Schier, A. Regev, Spatial reconstruction of single-cell gene expression data. Nat. Biotechnol. 33, 495–502 (2015). 25867923 10.1038/nbt.3192 PMC4430369

[85] S. Jin, C. F. Guerrero-Juarez, L. Zhang, I. Chang, R. Ramos, C. H. Kuan, P. Myung, M. V. Plikus, Q. Nie, Inference and analysis of cell-cell communication using CellChat. Nat. Commun. 12, 1088 (2021). 33597522 10.1038/s41467-021-21246-9 PMC7889871

[86] A. Riba, A. Oravecz, M. Durik, S. Jiménez, V. Alunni, M. Cerciat, M. Jung, C. Keime, W. M. Keyes, N. Molina, Cell cycle gene regulation dynamics revealed by RNA velocity and deep-learning. Nat. Commun. 13, 2865 (2022). 35606383 10.1038/s41467-022-30545-8 PMC9126911

[87] C. A. Schneider, W. S. Rasband, K. W. Eliceiri, NIH Image to ImageJ: 25 years of image analysis. Nat. Methods 9, 671–675 (2012). 22930834 10.1038/nmeth.2089 PMC5554542

